# Multi‐Omics Integration Reveals Incomplete Reactivation of Developmental Cell‐Cycle Programs in Adult Human Infarcted Hearts

**DOI:** 10.1111/cbdd.70337

**Published:** 2026-06-08

**Authors:** Jieran Lyu, Yuki Kuramoto, Jun Li, Jong‐Kook Lee, Kyoko Hidaka, Yasushi Sakata

**Affiliations:** ^1^ Department of Cardiovascular Medicine, Graduate School of Medicine The University of Osaka Osaka Japan; ^2^ Department of Cardiovascular Regenerative Medicine and Drug Discovery, Graduate School of Medicine The University of Osaka Osaka Japan; ^3^ Department of Pathophysiology of Heart Failure and Therapeutics National Cerebral and Cardiovascular Center Osaka Japan; ^4^ Open Innovation Center, Biobank National Cerebral and Cardiovascular Center Osaka Japan; ^5^ Faculty of Human Health, Sonoda University Amagasaki Hyogo Japan; ^6^ Center for Fundamental Education University of Kitakyushu Fukuoka Japan

**Keywords:** acute myocardial infarction, cardiac regeneration, cytokinesis, gene regulatory network inference, iPSC‐derived cardiomyocytes, multi‐omics integration

## Abstract

Adult human hearts show limited regeneration after acute myocardial infarction (AMI) despite a small subpopulation (~5%) of cardiomyocytes re‐entering the cell cycle. We integrated public single‐nucleus RNA‐seq, spatial transcriptomics, bulk RNA‐seq and ATAC‐seq datasets from human fetal hearts and adult infarcted hearts to investigate why cell‐cycle‐active cardiomyocytes fail to complete division. These cells were enriched in the infarct zone (IZ) and activated early cell‐cycle programs, but showed insufficient reactivation of late mitotic and cytokinesis execution machinery, particularly *ANLN* and *KIF18A*, and instead engaged stress‐adaptive programs. Multi‐omics filtering across expression, chromatin accessibility, and co‐expression/regulatory networks highlighted five developmental cell‐cycle‐associated genes with insufficient adult reactivation: *ANLN*, *KIF18A*, *MDK*, *RTKN2*, and *SOX11*. In human induced pluripotent stem cell‐derived cardiomyocytes, hypoxia suppressed most candidates, whereas *MDK* retained responsiveness to pro‐proliferative Wnt stimulation. These findings suggest that incomplete cytokinesis reactivation represents a key bottleneck for adult human cardiac regeneration, and nominate *MDK* as a tractable candidate for chemical biology and drug‐discovery efforts aimed at promoting cardiomyocyte cell‐cycle completion.

## Graphical abstract

Cross‐context integration of single‐nucleus, spatial, bulk transcriptomic, and chromatin accessibility data from human fetal and adult infarcted hearts reveals that infarct‐zone cardiomyocytes re‐enter the cell cycle but show insufficient reactivation of late mitotic and cytokinesis execution programs, suggesting that incomplete cytokinesis reactivation represents a key bottleneck for cardiac regeneration.

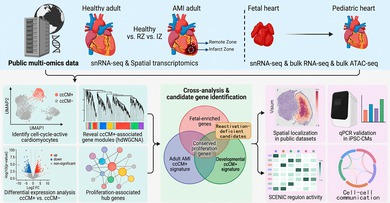

## 1 Introduction

Cardiovascular diseases cause over 18 million deaths globally each year, and acute myocardial infarction (AMI) remains a major cause of cardiac mortality (Martin et al. 2024). Despite significant advances in reperfusion therapy and pharmacological management, adult mammalian hearts respond to AMI through scar formation, rather than myocardial regeneration, leading to irreversible heart failure (Sadek and Olson 2020). The cardiac regenerative incapacity contrasts sharply with the robust cardiac regeneration observed in zebrafish (Lepilina et al. 2006) and neonatal mammals (Porrello et al. 2011), highlighting the urgent need to develop effective cardiac regenerative therapies.

The proliferative capacity of human cardiomyocytes undergoes a dramatic developmental decline. During fetal development, cardiomyocytes proliferate robustly, expanding from thousands to billions of cells (Mollova et al. 2013). This capacity declines rapidly after birth, with adult cardiomyocyte turnover rates estimated to be < 1% annually (Mollova et al. 2013). Notably, Meckert et al. (2005) demonstrated that 7–13 days post‐infarction, 11.61% ± 6.94% of peri‐infarct cardiomyocytes express the proliferation marker MKI67, yet these cells undergo endomitosis within preserved nuclear envelopes rather than true cell division, resulting in polyploidization (Meckert et al. 2005). This phenomenon suggests that cell‐cycle re‐entry mechanisms exist; however, the execution programs may remain incomplete, representing a critical barrier to cardiac regeneration.

Although neonatal mammalian injury models have provided important insights into cardiac regeneration (Costa et al. 2022; Liu et al. 2021), species‐specific differences in the timing and regulation of postnatal cell‐cycle exit limit direct extrapolation to the human context. The neonatal regenerative window is transient, and human cardiomyocytes undergo a particularly rapid decline in proliferative capacity during postnatal development (Mollova et al. 2013), rendering human‐specific studies essential. Understanding the molecular mechanisms underlying human cardiac regenerative failure requires a direct comparison of fetal and adult cardiomyocyte cell‐cycle programs at single‐nucleus resolution. However, the scarcity of human cardiac tissue severely limits such investigations. A systematic search of PubMed and Web of Science databases (January 2005 to March 2026) identified, to our knowledge, no published study that has systematically compared human developmental and adult post‐AMI cardiomyocyte cell‐cycle programs across matched single‐nucleus, spatial, bulk transcriptomic, and chromatin accessibility modalities.

The recent release of high‐quality public datasets from multiple independent cohorts provides an unprecedented opportunity to address this knowledge gap (Kuppe et al. 2022; Litvinukova et al. 2020; Sim et al. 2021). These multi‐omics datasets encompassing human fetal heart development and adult AMI tissues, including single‐nucleus RNA‐seq, spatial transcriptomics, and ATAC‐seq data, have established tissue‐level cellular atlases. However, a systematic cross‐modal comparison of cardiomyocyte cell‐cycle programs between developmental and adult post‐injury contexts has not been performed. The integrative analysis of these public datasets overcomes individual sample limitations while providing the statistical power necessary for identifying robust biological signals.

Herein, we present a single‐nucleus‐resolution comparison of cardiomyocyte cell‐cycle programs between human fetal (Sim et al. 2021) and adult AMI hearts (Kuppe et al. 2022). Through an integrative analysis of multiple public datasets, we identified cell‐cycle‐active cardiomyocyte subpopulations in adult AMI hearts and revealed their enrichment in the infarct zone (IZ). In‐depth analysis demonstrated that although these cell‐cycle‐active cardiomyocytes expressed early cell‐cycle genes, they showed insufficient reactivation of critical late mitotic and cytokinesis execution genes, particularly *ANLN* (Engel et al. 2006) and *KIF18A* (Mayr et al. 2007), which form coordinated co‐expression networks during fetal development but show markedly insufficient reactivation in adult AMI. Building on these computational findings, we examined the expression dynamics of candidate genes in human iPSC‐derived cardiomyocytes under ischemic and pro‐proliferative conditions. Our integrative analytical framework provides molecular insights into incomplete cell‐cycle program reactivation in adult human infarcted hearts and identifies MDK (midkine; Horiba et al. 2006), which maintains responsiveness to pro‐proliferative signals under hypoxia, representing a candidate for future investigations in cardiac regeneration.

## 2 Results

### 2.1 Integrated Multi‐Omics Framework Reveals Context‐Dependent Cardiomyocyte Cell‐Cycle Programs

**FIGURE 1:**
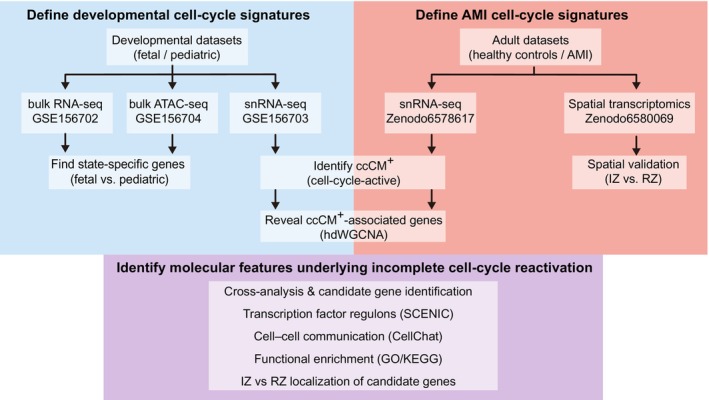
Multi‐omics integration framework for characterizing incomplete cell‐cycle program reactivation in adult infarcted hearts. Developmental datasets (left) comprising snRNA‐seq, bulk RNA‐seq, and ATAC‐seq were used to define fetal and pediatric cell‐cycle signatures. Adult datasets (right) comprising snRNA‐seq and spatial transcriptomics were analyzed in parallel. In both contexts, cardiomyocytes were classified into cell‐cycle‐active (ccCM^+^) and cell‐cycle‐inactive (ccCM^−^) states, and co‐expression networks were constructed using hdWGCNA. Cross‐dataset integration (bottom) identified developmental programs with insufficient adult reactivation, which were further characterized through regulatory network inference (SCENIC), intercellular communication analysis (CellChat), and functional enrichment.

To identify the molecular barriers underlying adult human cardiac regenerative failure, we established an integrative analytical framework to compare cardiomyocyte cell‐cycle programs between the developmental and disease contexts (Figure 1). For developmental analysis, we integrated single‐nucleus RNA‐seq (snRNA‐seq) data spanning the fetal (14 weeks post‐conception) to pediatric (14 years) stages, complemented by bulk RNA‐seq and ATAC‐seq datasets, to capture both transcriptional and epigenetic signatures. For adult analysis, we examined snRNA‐seq data from control and AMI hearts, with spatial transcriptomics providing regional validation.

Within each snRNA‐seq dataset, cardiomyocytes were stratified into cell‐cycle‐active (ccCM^+^) and cell‐cycle‐inactive (ccCM^−^) states using canonical proliferation markers and AUCell scoring (see Section 4). This classification enabled independent differential expression and high‐dimensional weighted gene co‐expression network analyses (hdWGCNA) within each context, revealing the distinct regulatory networks underlying cell‐cycle‐active states, which formed the basis for identifying programs that fail to reactivate in the adult context. Cross‐dataset integration identified developmental proliferation signatures that failed to reactivate during adult AMI. By comparing genes robustly expressed in fetal ccCM^+^ with those showing insufficient upregulation in adult AMI ccCM^+^, we identified candidate molecular barriers to cytokinesis. These candidates were subsequently characterized through regulatory network inference (SCENIC), cell–cell communication analysis (CellChat), spatial transcriptomics, and quantitative PCR experiments in human iPSC‐derived cardiomyocytes (iPSC‐CMs).

### 2.2 Cell‐Cycle‐Active Cardiomyocytes Are Enriched in Adult IZ

**FIGURE 2:**
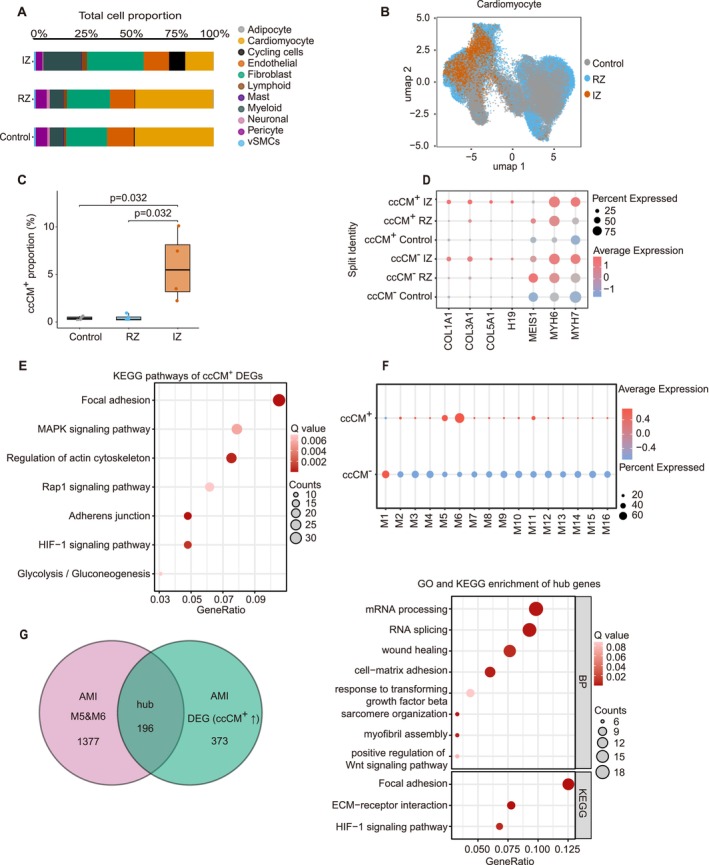
Identification and characterization of cell‐cycle‐active cardiomyocytes in adult infarct zone. (A) Cell‐type composition across conditions. (B) UMAP visualization of cardiomyocytes colored by tissue region. (C) Per‐donor ccCM^+^ proportion (*n* = 4 control, *n* = 5 RZ, *n* = 4 IZ). Kruskal–Wallis test with post hoc Mann–Whitney U tests (Holm‐adjusted). (D) Dot plot of selected dedifferentiation and stress‐adaptation markers across regions and cell‐cycle states. Dot size indicates percentage of expressing cells; color intensity represents average expression level. (E) KEGG pathway enrichment of ccCM^+^ differentially expressed genes. (F) Module–trait correlation heatmap from hdWGCNA. Modules M5 and M6 show positive associations with the ccCM^+^ state. (G) Venn diagram defining AMI hub genes as the intersection of M5/M6 module genes and ccCM^+^‐upregulated DEGs (right: Functional enrichment of hub genes).

To determine whether cell‐cycle‐active cardiomyocytes (ccCM^+^) exist in adult human hearts following injury, we analyzed snRNA‐seq data from adult controls and AMI samples. Cellular composition analysis revealed a marked redistribution of major cardiac cell types among the healthy control myocardium, remote zone (RZ), and IZ (Figure 2A, Figure S2A). As expected, the IZ demonstrated substantial cardiomyocyte depletion accompanied by expansion of fibroblasts and immune cells. Sub‐clustering analysis of all cardiomyocytes annotated by sample origin (control, RZ, and IZ) revealed considerable transcriptional heterogeneity across regions (Figure 2B).

To identify cardiomyocytes with cell‐cycle activity within the surviving myocardium, we scored eight canonical proliferation markers (*AURKB*, *BUB1*, *CCNB1*, *CDCA8*, *CDK1*, *MKI67*, *PLK1*, and *TOP2A*) using AUCell (Figure S1A; see Section 4). A unified threshold of 0.03 was applied to classify ccCM^+^ and ccCM^−^, selected as the lowest threshold recapitulating the expected developmental hierarchy of cardiomyocyte proliferative capacity (Figure S1B,C; see Section 4). Regional analysis revealed that IZ‐derived cardiomyocytes exhibited significantly higher cell‐cycle marker expression than those from the RZ and control samples (Figure 2B, Figure S2B,C). Quantitative assessment across individual donors demonstrated that the ccCM^+^ proportion was significantly enriched in the IZ (median 5.48%) compared to both the RZ (0.25%) and control samples (0.38%) (Kruskal–Wallis *H* = 7.74, *p* = 0.021; post hoc Mann–Whitney *U* tests with Holm adjustment, IZ vs. RZ, *p* = 0.032; IZ vs. control, *p* = 0.032), whereas no significant difference was observed between the RZ and control groups (*p* = 0.905) (Figure 2C). This regional enrichment, specifically within the IZ, suggests that local injury signals trigger cell‐cycle programs in the surviving cardiomyocytes. Dot plot analysis confirmed the upregulation of all eight proliferation markers in ccCM^+^ versus ccCM^−^ across regions (Figure S2D), and traditional S‐phase and G2/M module scores independently confirmed the elevated cell‐cycle activity in ccCM^+^ (Figure S2E).

In addition to cell‐cycle activation, IZ cardiomyocytes exhibited features consistent with partial dedifferentiation and stress adaptation. Expression profiling across regions and cell‐cycle states revealed upregulation of the fetal gene *H19* (He et al. 2022; Kuwahara et al. 2012), structural remodeling genes (*COL1A1*, *COL3A1*, and *COL5A1*), and altered contractile isoform ratios (*MYH6*/*MYH7*), along with reduced expression of *MEIS1*, a transcription factor associated with postnatal cell‐cycle exit (Mahmoud et al. 2013), in IZ cardiomyocytes (Figure 2D). Functional enrichment analysis of genes upregulated in ccCM^+^ compared to those in ccCM^−^ revealed that Gene Ontology terms highlighted cytoskeletal reorganization and stress responses, particularly in response to decreased oxygen levels (Figure S2F). Kyoto Encyclopedia of Genes and Genomes (KEGG) pathway analysis demonstrated prominent activation of HIF‐1 signaling and metabolic reprogramming pathways, including glycolysis/gluconeogenesis, along with structural reorganization pathways, such as focal adhesion and actin cytoskeleton regulation (Figure 2E). The predominance of the hypoxia‐responsive and metabolic adaptation pathways indicates that ccCM^+^ respond primarily to an ischemic microenvironment. Collectively, these findings indicate that adult cardiomyocytes can initiate cell‐cycle programs following injury but predominantly activate stress‐adaptive programs, with limited enrichment of late mitotic or cytokinesis‐specific pathways.

### 2.3 Co‐Expression Network Analysis Identifies Proliferation‐Associated Gene Modules in Adult IZ

Having identified ccCM^+^ enriched in IZ, we next employed hdWGCNA to dissect the underlying gene regulatory networks and identify the core genes driving the cell‐cycle‐active state. We performed hdWGCNA on adult cardiomyocytes encompassing the control, RZ, and IZ samples. Network construction using a soft‐thresholding power of *β* = 8 achieved an approximate scale‐free topology (*R* ^2^ = 0.80), and dynamic tree cutting identified 16 distinct co‐expression modules ranging from 50 to 1500 genes (Figure S3A–C).

To determine the modules that were specifically associated with the cell‐cycle‐active state, we mapped module activity across the cardiomyocyte landscape using UCell scores. This analysis revealed that modules M5 and M6 specifically colocalized with the ccCM^+^ manifold in the UMAP space (Figure S3D). Module‐trait correlation analysis confirmed significant positive associations between modules M5 and M6 and the cell‐cycle‐active state (Figure 2F). Network topology analysis revealed distinct hub gene signatures within each module (Figure S3E).

To characterize the biological significance of these proliferation‐associated modules, we performed functional enrichment analysis of the combined M5 and M6 gene sets (Figure S3F). This analysis revealed coordinated programs spanning muscle organ development, myofibril assembly, RNA splicing regulation, and wound healing. KEGG pathways prominently featured focal adhesion, regulation of the actin cytoskeleton, and extracellular matrix (ECM)‐receptor interactions, indicating that these modules capture both structural reorganization and matrix remodeling processes that are characteristic of the injury response.

We then integrated the hdWGCNA modules with differential expression analysis to define the core gene set that drives the cell‐cycle response. This integration identified 196 AMI hub genes representing the intersection of the M5/M6 module genes and upregulated genes in ccCM^+^ (Figure 2G). Functional enrichment analysis of this refined hub gene set revealed processes such as wound healing, cell‐matrix adhesion, and response to transforming growth factor beta (TGF‐*β*). Notably, the analysis also identified positive regulation of Wnt and HIF‐1 signaling pathways, suggesting coordinated activation of developmental proliferation programs and hypoxia response mechanisms (Figure 2G). Additional enriched pathways included focal adhesion and ECM‐receptor interactions, further highlighting the importance of matrix remodeling in the cell‐cycle response. These AMI hub genes represent network‐centered injury‐activated genes that coordinate stress adaptation and partial cell‐cycle program activation.

### 2.4 Developmental Cell‐Cycle Programs Defined by Multi‐Omics Integration

**FIGURE 3:**
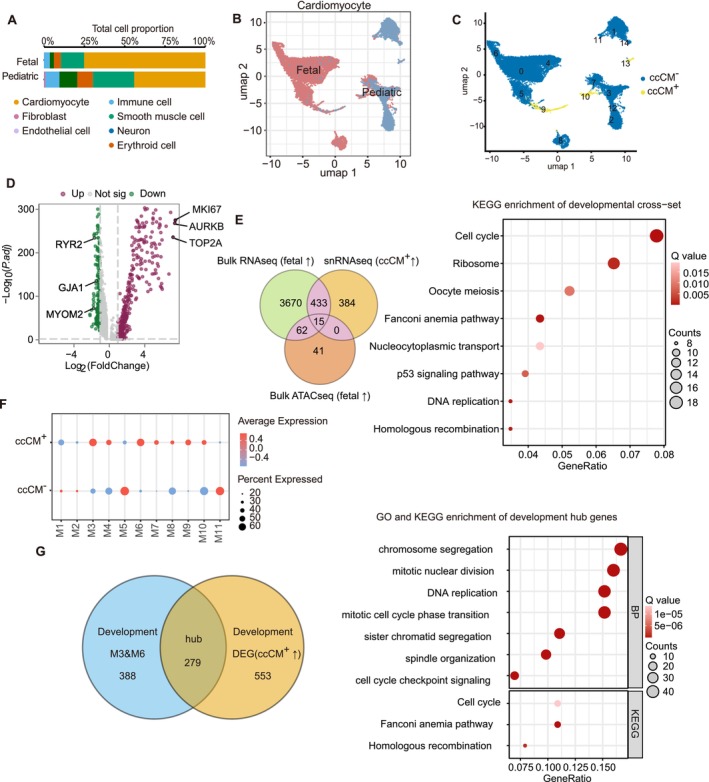
Multi‐omics characterization of cell‐cycle‐active cardiomyocytes during cardiac development. (A) Cell‐type composition across developmental stages. (B) UMAP visualization of cardiomyocytes by sample origin (fetal and pediatric). (C) Left: Cardiomyocyte subclustering into 15 transcriptionally distinct groups. Right: CcCM^+^ and ccCM^−^ classification. (D) Volcano plot of differential gene expression between ccCM^+^ and ccCM^−^ in developmental hearts. Representative proliferation markers and maturation markers are labeled. (E) Venn diagram showing overlap among fetal‐upregulated genes identified across three data modalities (bulk RNA‐seq, snRNA‐seq, and ATAC‐seq), defining the developmental cross‐validated gene set. Right: KEGG pathway enrichment of the cross‐validated set. (F) Module–trait correlation heatmap from hdWGCNA. Modules M3 and M6 show positive correlations with the ccCM^+^ state. (G) Venn diagram defining developmental hub genes as the intersection of M3/M6 module genes and ccCM^+^‐upregulated DEGs (right: GO and KEGG enrichment of hub genes).

To establish a reference for normal cardiac proliferation and evaluate whether adult injury responses recapitulate developmental mechanisms, we analyzed snRNA‐seq data spanning fetal to pediatric human hearts. The analysis revealed distinct changes in cellular composition during cardiac maturation, with major cell types, including cardiomyocytes, fibroblasts, endothelial cells, smooth muscle cells, immune populations, neurons, and erythroid cells, identified across developmental stages (Figure S4A). Notably, compositional analysis demonstrated that cardiomyocytes constituted a higher proportion of total cells in fetal hearts than in pediatric hearts, while non‐myocyte populations increased during postnatal maturation (Figure 3A). This shift reflects the transition from a proliferative to a mature cardiac environment.

Using the same AUCell‐based approach applied to adult AMI samples, we identified cell‐cycle‐active cardiomyocytes in developmental hearts (Figure S1A–C). Within the cardiomyocyte compartment, clustering revealed a clear separation between fetal and pediatric samples, reflecting distinct transcriptional states (Figure 3B, Figure S1A). Subclustering identified 15 transcriptionally distinct cardiomyocyte groups, with subclusters 9, 10, and 14 corresponding to cell‐cycle‐active states that showed focal expression of canonical proliferation markers and were predominantly fetal‐derived (Figure 3C, Figure S4B). The concentration of ccCM^+^ in fetal samples contrasts sharply with their scattered distribution in adult AMI hearts, highlighting the fundamental differences between developmental proliferation and injury‐induced regeneration attempts.

Differential expression analysis comparing ccCM^+^ versus ccCM^−^ across all developmental stages identified molecular programs distinguishing developmental cell‐cycle‐active states. ccCM^+^ showed upregulation of canonical cell‐cycle regulators, including *AURKB*, *MKI67*, and *TOP2A* (Figure 3D). In contrast, ccCM^−^ exhibited enrichment of maturation markers, including *MYOM2*, *GJA1*, and *RYR2*, indicating an inverse relationship between proliferation and terminal differentiation.

To characterize the developmental cell‐cycle landscape beyond single‐nucleus resolution, we performed parallel analyses of bulk RNA‐seq and ATAC‐seq data, comparing fetal and pediatric samples (Figure S4C). These analyses yielded 8646 differentially expressed genes (4185 upregulated in fetal tissues) and 371 differential chromatin accessibility regions (118 with increased accessibility in fetal tissues), demonstrating robust stage‐dependent transcriptional and epigenetic changes. Differential expression analysis using snRNA‐seq identified 832 genes that were upregulated in developmental ccCM^+^. The integration of bulk datasets with single‐nucleus expression patterns across the three data modalities defined a cross‐validated gene set of 510 genes, comprising only those supported by at least two independent data types (Figure 3E).

Functional enrichment of this developmental cross‐validated set confirmed coherent cell‐cycle and genome maintenance programs (Figure 3E, Figure S4D). KEGG pathways prominently featured the cell‐cycle and DNA replication processes, whereas Gene Ontology terms emphasized nuclear division and chromosome segregation. These convergent signatures across multiple data modalities establish a comprehensive molecular blueprint for normal cardiac proliferation.

### 2.5 Shared and Distinct Cell‐Cycle Programs Between Developmental and Adult Contexts

To establish a reference cell‐cycle network architecture for comparison with adult AMI, we performed parallel hdWGCNA analysis of developmental cardiomyocytes. Network construction with a soft threshold of *β* = 8 achieved scale‐free topology (*R* ^2^ = 0.80), yielding 11 distinct modules through dynamic tree cutting (Figure S5A–C). Module activity mapping revealed that modules M3 and M6 were specifically enriched in cell‐cycle‐active populations (Figure S5D), and module‐trait correlation analysis confirmed significant positive associations after multiple testing corrections (Figure 3F). Notably, M6 contained robust cell‐cycle regulatory genes, with hub genes including *ANLN*, *KIF11*, *BUB1B*, and *CIT* representing critical mitotic and cytokinesis factors (Figure S5E).

To define the core developmental cell‐cycle genes, we integrated module gene lists with differential expression and identified developmental hub genes as the intersection of ccCM^+^‐associated module genes and ccCM^+^‐upregulated DEGs (Figure 3G). Functional enrichment analysis demonstrated that these developmental hub genes coordinated complete cell‐cycle execution, with Gene Ontology and KEGG analyses emphasizing the mitotic cell‐cycle, chromosome segregation, nuclear division, and DNA replication pathways (Figure 3G). Notably, this developmental program included coordinated expression of late mitotic and cytokinesis execution factors within M6, such as *ANLN*, *KIF11*, and *BUB1B* (Figure S5E). In contrast, adult AMI hub genes enriched stress‐adaptive and structural remodeling pathways without activation of these cytokinesis modules (Figure 2G vs. Figure 3G).

**FIGURE 4:**
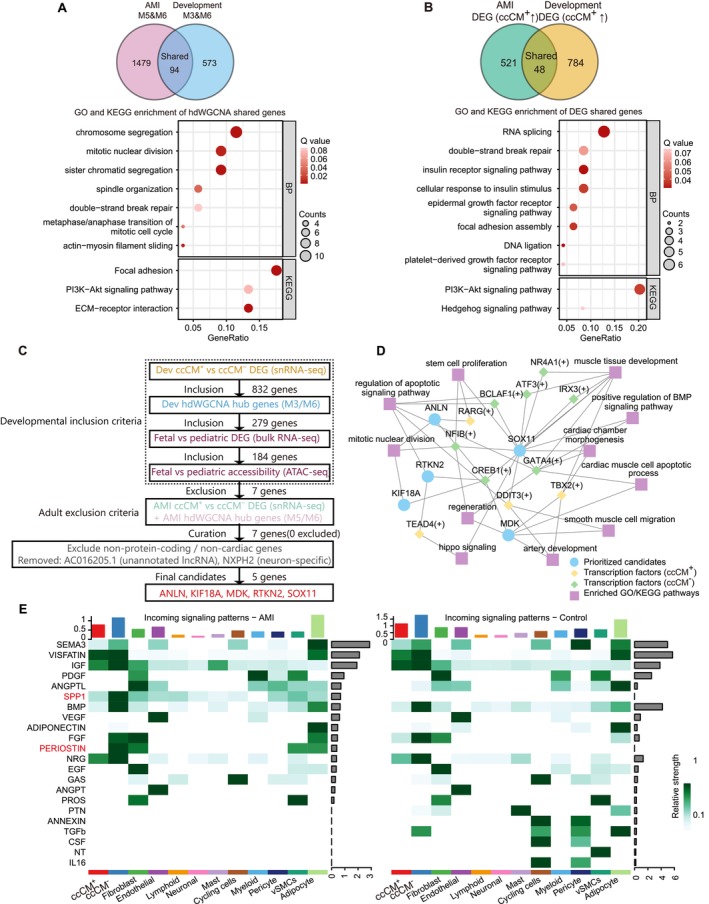
Cross‐context comparison of cell‐cycle programs and regulatory networks. (A) Venn diagram showing overlap between AMI (M5/M6) and developmental (M3/M6) hdWGCNA module genes, defining hdWGCNA‐shared genes (bottom: GO and KEGG enrichment). (B) Venn diagram showing overlap between AMI and developmental ccCM^+^‐upregulated DEGs, defining DEG‐shared genes (bottom: GO and KEGG enrichment). (C) Sequential multi‐omics filtering flowchart for candidate gene identification. Developmental inclusion criteria (top) and adult exclusion filters were applied as described in Methods; gene counts at each step are indicated. (D) Network visualization integrating the five candidate genes with SCENIC‐identified transcription factors and enriched pathways in adult hearts. Node shapes distinguish candidate genes, transcription factors associated with ccCM^+^ or ccCM^−^ states, and enriched GO/KEGG terms (see inset legend). (E) CellChat pathway‐level incoming signaling strength to each cell type in adult AMI (left) versus controls (right). Color intensity denotes relative signaling strength.

Building on these context‐specific analyses, we systematically identified shared cell‐cycle programs between the developmental and adult contexts using two complementary approaches. First, we identified hdWGCNA‐shared genes representing module‐level overlap between the developmental and AMI datasets, which captured network‐coordinated functional units active in both contexts (Figure 4A). These network‐level shared genes were enriched for core cell‐cycle machinery, including chromosome and DNA replication/repair programs and spindle organization. Second, we identified DEG‐shared genes representing direct transcriptional overlap, defining specific effector genes upregulated in ccCM^+^ regardless of the context (Figure 4B). These shared expression‐level genes were enriched for cellular signaling and structural remodeling pathways, including RNA splicing, insulin/IGF receptor signaling, and focal adhesion assembly. The distinction between network‐level and expression‐level overlaps provides complementary insights; the former identifies a conserved regulatory architecture, whereas the latter identifies specific effector genes that are consistently activated across contexts.

### 2.6 Transcriptional Regulatory Networks Governing Cell‐Cycle‐Active Cardiomyocyte States

A cross‐context comparison revealed that, while developmental and adult ccCM^+^ share certain core programs (Figure 4A–B), many developmental proliferation genes fail to reactivate following adult AMI. Identifying the specific genes that remain silent despite injury‐induced cell‐cycle re‐entry would pinpoint molecular positions at which the adult program stalls, thereby defining candidate targets for strategies aimed at enabling cardiomyocyte division completion. To identify developmental cell‐cycle genes with insufficient adult reactivation, we applied sequential multi‐omics filtering starting from all 832 developmental ccCM^+^‐upregulated genes, requiring hub gene status within developmental hdWGCNA modules, fetal‐enriched expression (bulk RNA‐seq), and fetal‐accessible chromatin (ATAC‐seq), combined with exclusion of genes showing reactivation in adult AMI ccCM^+^ (Figure 4C; see Section 4 for detailed criteria). This filtering progressively narrowed the candidate pool from 832 to 7 genes; none were excluded by the adult AMI filters, confirming their insufficient reactivation. After curation, five candidate genes remained: *ANLN*, *KIF18A*, *MDK*, *RTKN2*, and *SOX11*.

To understand the regulatory basis for this incomplete reactivation, we characterized the transcriptional networks governing cell‐cycle‐active states across contexts using SCENIC analysis. Network reconstruction of adult hearts revealed that the identified candidates converged on key developmental pathways, including Hippo signaling, stem cell proliferation, cardiac chamber morphogenesis, and artery development (Figure 4D). In contrast, SCENIC analysis of developmental cardiomyocytes revealed a distinct regulatory architecture centered on proliferation‐enabling pathways, with E2F family transcription factors orchestrating mitotic cell‐cycle phase transitions alongside regulons involved in tissue regeneration and developmental signaling (Figure S6A).

A comparison of regulon activities between contexts identified shared and context‐specific regulatory programs. Four transcription factors, *CEBPB*, *FLI1*, *JUND*, and *MYB*, showed consistently elevated activity in ccCM^+^ in both developmental and AMI contexts, with the *FLI1* regulon demonstrating particularly robust enrichment in both settings (Figure S6B–D).

However, downstream targets and co‐activated programs differed markedly between contexts. Developmental ccCM^+^ engaged in comprehensive cell‐cycle modules orchestrated by the E2F family members, *MYC* and *MYBL1/2*, along with chromatin modifiers such as *EZH2* and *SMARCA4* (Figure S6C). In contrast, adult AMI ccCM^+^ preferentially activated stress‐responsive regulons, reflecting adaptation to hypoxia and proteotoxic stress, rather than productive division (Figure S6B). This shift from growth‐oriented to stress‐responsive transcriptional programs may explain the limited regenerative capacity of adult hearts.

### 2.7 Intercellular Signaling Landscapes Differ Between Developmental and Adult Contexts

While transcriptional programs provide intrinsic machinery for proliferation, extrinsic signals from the cardiac microenvironment critically influence whether cardiomyocytes can complete their cell division. Therefore, we used CellChat to map intercellular communication networks and understand how paracrine signaling contributes to the differential proliferative capacity across contexts.

In the fetal heart, ccCM^+^ receive diverse signaling inputs from multiple cellular sources. Fibroblasts, endothelial cells, smooth muscle cells, immune populations, and neurons collectively provided FGF, IGF, NRG, WNT, and BMP signals, creating a redundant growth‐supportive environment (Figure S7A). At ligand‐receptor resolution, developmental ccCM^+^ engaged multiple growth factor receptor systems from diverse cellular origins (Figure S7B). The transition to the pediatric heart showed uniformly diminished pathway activity across all major signaling axes, coinciding with the loss of proliferative capacity.

At the intercellular signaling level, adult AMI partially reactivated select growth‐supportive pathways that are active during fetal development, including BMP and NRG signaling, while simultaneously engaging in injury‐specific programs (Figure 4E). PERIOSTIN and SPP1 pathways associated with matrix remodeling and fibrosis, which are minimal in healthy hearts, were prominently activated. Regional analysis revealed that these injury‐responsive pathways, along with NRG, showed stronger activity in the IZ than in the RZ (Figure S7C). At ligand‐receptor resolution, the adult AMI signaling repertoire was notably more restricted than during development: select growth‐promoting interactions, such as NRG2‐ERBB4 and IGF1‐IGF1R, showed partial reactivation, but injury‐associated and stress‐responsive interactions predominated (Figure S7D).

Together with the shift from growth‐oriented to stress‐responsive transcriptional programs, this altered signaling landscape indicates that the adult IZ fails to recapitulate the growth‐supportive microenvironment present during fetal development. The combination of cell‐autonomous regulatory barriers and non‐permissive paracrine signaling suggests a dual constraint on cardiac regeneration in adults.

### 2.8 Spatial Transcriptomics Validates Partial Reactivation of Candidate Genes in Adult IZ

Having identified cell‐autonomous transcriptional barriers and altered paracrine signaling as potential constraints on regeneration, we sought to validate the expression patterns of these five candidate genes in the adult infarct microenvironment. To examine tissue‐level expression patterns, we analyzed 10× Visium spatial transcriptomic data from the same AMI cohort used for the snRNA‐seq analysis (Figure S8A and Table S2). The analysis confirmed the expected cellular redistribution, with cardiomyocyte depletion and concurrent stromal and immune cell expansion in the IZ compared to the RZ (Figure S8B,C).

**FIGURE 5:**
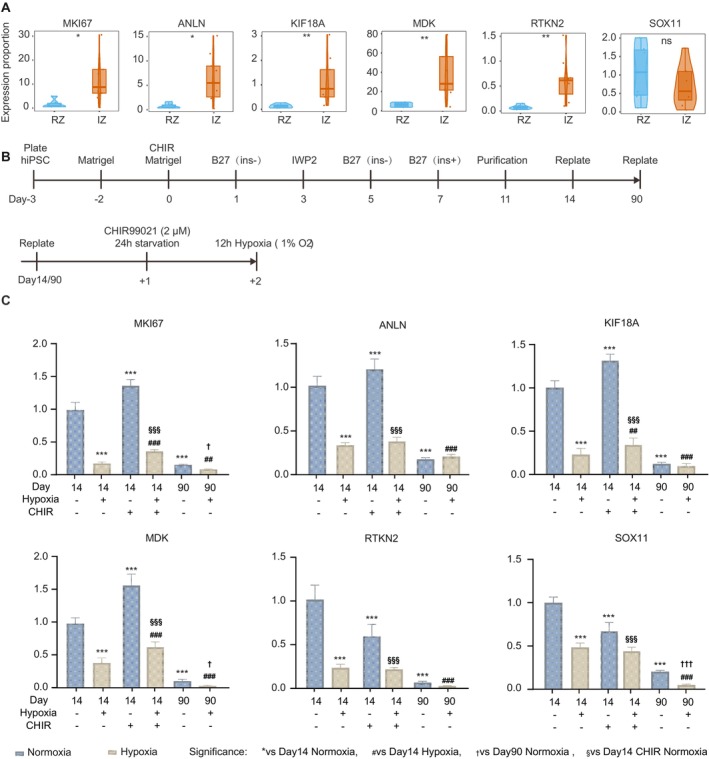
Spatial validation and experimental characterization of candidate genes. (A) Proportion of spots expressing MKI67 and each candidate gene in IZ versus RZ from 10× Visium spatial transcriptomic data. Wilcoxon rank‐sum tests with Benjamini–Hochberg correction. (B) Experimental timeline for iPSC‐CM differentiation and treatment conditions. (C) qPCR analysis of candidate gene expression in iPSC‐CMs at Day 14 (immature) and Day 90 (mature) under normoxic and 12‐h hypoxic conditions. Day 14 cardiomyocytes were additionally treated with or without CHIR99021 (2 μM) under both oxygen conditions. Data represent mean ± SEM (*n* = 8 biological replicates). Two‐way ANOVA with Tukey's post hoc test; significance symbols are defined in the panel. *P < 0.05, **P < 0.01, ***P < 0.001 vs Day14 Normoxia; ^#^ *P* < 0.05, ^##^ *P* < 0.01, ^###^ *P* < 0.001 vs Day14 Hypoxia; ^†^ *P* < 0.05, ^††^ *P* < 0.01, ^†††^ *P* < 0.001 vs Day90 Normoxia; ^§^ *P* < 0.05, ^§§^ *P* < 0.01, ^§§§^ *P* < 0.001 vs Day14 CHIR Normoxia.

To assess the regional expression differences, we quantified the proportion of spots expressing each candidate gene. The proliferation marker *MKI67* showed significantly elevated expression in IZ, validating our analytical approach. Similarly, four of the five candidates, *ANLN*, *KIF18A*, *MDK*, and *RTKN2*, exhibited significantly higher expression proportions in the IZ than in the RZ, whereas *SOX11* showed no significant regional differences (Figure 5A; two‐sided Wilcoxon rank‐sum test with Benjamini–Hochberg correction, *p* < 0.05 for each significant comparison).

Although these candidates showed coordinated elevation with MKI67 in the IZ, confirming their association with cell‐cycle programs, their reactivation magnitude remained substantially below developmental levels as determined by snRNA‐seq analysis. This partial rather than complete reactivation is consistent with the regulatory barriers identified in our transcriptional and signaling analyses.

### 2.9 Expression Dynamics of Candidate Genes in Human iPSC‐Derived Cardiomyocytes

We used human iPSC‐CMs to characterize the expression dynamics of the identified candidates under conditions modeling the ischemic microenvironment and pro‐proliferative signaling. We examined expression in Day 14 (immature) versus Day 90 (mature) iPSC‐CMs under normoxia or 12‐h hypoxia. In Day 14 cardiomyocytes, we additionally tested CHIR99021 treatment (2 μM), a Wnt pathway activator known to promote cardiomyocyte proliferation (Maas et al. 2021) (Figure 5B). Combined nutrient deprivation and hypoxia (1% O_2_) were used to model the ischemic microenvironment of AMI (Hakli et al. 2021; Snyder et al. 2024). Hereafter, hypoxia refers to this combined ischemic condition unless otherwise specified. Wnt activation was tested based on our earlier finding that Wnt signaling is enriched in AMI ccCM^+^ (Figure 2G). CHIR99021‐treated Day 14 cardiomyocytes served as an immature, Wnt‐activated comparator with retained proliferative competence.

Hypoxia robustly induced the canonical HIF‐1 target genes *BNIP3* and *SLC2A1*, thus validating the experimental conditions (Figure S9). Under hypoxic conditions, *MKI67* and all five candidates showed significantly higher expression on Day 14 than on Day 90, indicating that immature cardiomyocytes retained higher proliferative gene expression even under ischemic stress (Figure 5C).

Hypoxic exposure produces different effects depending on the state of cardiomyocyte maturation. In immature Day 14 cardiomyocytes, hypoxia uniformly suppressed all examined genes (*p* < 0.001 for each). In contrast, mature Day 90 cardiomyocytes showed heterogeneous responses. *MKI67*, *MDK*, and *SOX11* were significantly suppressed, whereas *ANLN*, *KIF18A*, and *RTKN2* showed no significant change, consistent with their already low baseline expression at this maturation stage. This selective hypoxic sensitivity suggests that proliferation‐associated genes are differentially regulated during cardiomyocyte maturation.

Next, we tested whether CHIR99021 treatment could modulate the expression of candidate genes. Under normoxia, CHIR significantly enhanced the expression of *ANLN*, *KIF18A*, *MDK*, and *MKI67* in Day 14 cardiomyocytes (*p* < 0.001 for each), while suppressing *RTKN2* and *SOX11* (*p* < 0.001 for both). Under hypoxic conditions, Day 14 cardiomyocytes showed markedly heterogeneous responsiveness to CHIR. *MDK* and *MKI67* demonstrated robust CHIR‐mediated expression recovery (*p* < 0.001 for both), *KIF18A* showed modest recovery (*p* < 0.01), whereas *ANLN*, *RTKN2*, and *SOX11* remained unresponsive to CHIR stimulation. These divergent responses suggest that proliferation‐associated genes are governed by distinct regulatory mechanisms. *RTKN2* and *SOX11* were suppressed by Wnt activation, indicating that their re‐expression in the context of cardiac regeneration would require alternative, Wnt‐independent strategies.

These findings established that the identified candidates, while sharing developmental expression patterns and associations with cell‐cycle programs, exhibited distinct regulatory properties under stressful conditions. The suppression of most candidates under hypoxia, even with Wnt activation, is consistent with their insufficient reactivation in adult AMI despite the presence of proliferative signals. Among these candidates, *MDK* emerged as particularly notable for maintaining responsiveness to pro‐proliferative signals even under hypoxic stress, warranting further investigation as a candidate regulator of cardiac regeneration.

## 3 Discussion

We analyzed publicly available multi‐omics datasets from multiple independent cohorts to systematically characterize cardiomyocyte cell‐cycle programs during human cardiac development and adult myocardial infarction. Our integrative analysis revealed that adult hearts show incomplete cell‐cycle program reactivation following injury, characterized by expression of cell‐cycle markers without proportional activation of late mitotic and cytokinesis execution programs. Through a systematic comparison of developmental and adult datasets, we identified five candidate genes, *ANLN*, *KIF18A*, *MDK*, *RTKN2*, and *SOX11*, whose robust expression in fetal hearts failed to sufficiently reactivate following AMI. We examined the expression patterns of these candidates in human iPSC‐derived cardiomyocytes, demonstrating differential responsiveness to hypoxia and Wnt activation, with *MDK* showing maintenance of responsiveness to pro‐proliferative signals under stress conditions.

The enrichment of ccCM^+^ specifically within the adult IZ provides important insights into the human cardiac injury response. These cells express canonical proliferation markers, including *AURKB*, *MKI67*, and *TOP2A*, and exhibit features suggestive of partial dedifferentiation, such as fetal gene reactivation (*H19*) and sarcomeric alterations. We classified ccCM^+^ using AUCell (Aibar et al. 2017) with eight core proliferation markers rather than broader S or G2/M phase‐specific gene sets. In adult cardiomyocytes, cell‐cycle gene expression is extremely sparse, rendering large phase‐specific gene sets unreliable for per‐cell classification; a focused marker panel with rank‐based aggregate scoring more robustly detects any level of cell‐cycle activity. The continuous rather than bimodal distribution of AUCell scores aligns with evidence that *MKI67* expression reflects a graded signal of cell‐cycle engagement (Miller et al. 2018). In the absence of a natural bimodal boundary, a threshold‐based classification serves as an operational tool for downstream categorical analyses, the robustness of which was confirmed through threshold sensitivity analyses (Figure S1B) and threshold‐free continuous comparisons (Figure S1D). The unified threshold of 0.03 was the lowest value at which the expected developmental hierarchy—fetal exceeding pediatric ccCM^+^ proportion (Mollova et al. 2013)—was correctly recapitulated. The core finding of regional enrichment (IZ > RZ > Control) was stable across all tested thresholds (0.01–0.10), and threshold‐free comparison of per‐donor mean scores independently confirmed elevated cell‐cycle activity in the IZ (Kruskal–Wallis, *p* = 0.021; Figure S1D).

Comparison of ccCM^+^‐associated co‐expression modules between developmental and adult contexts (Figure 2G vs. Figure 3G) revealed that the nature of cell‐cycle re‐entry differs fundamentally between these settings. Despite the expression of cell‐cycle markers, AMI hub genes lack late‐phase cell‐cycle executors and instead enrich stress response pathways. Consistent with our findings, Hume et al. (2026) recently reported that cardiomyocyte mitotic events increase following human MI, with protein‐level evidence of progressive attrition from phospho‐histone H3 positivity through Aurora kinase B to midbody protein MKLP1 expression, indicating that most cell‐cycle re‐entry events terminate before cytokinesis completion. This independent observation aligns with previous histological evidence of peri‐infarct cardiomyocyte *MKI67* expression accompanied by endomitosis rather than productive division (Kirillova et al. 2021; Meckert et al. 2005), and supports our transcriptomic finding that adult AMI ccCM^+^ activate early cell‐cycle genes without engaging late mitotic and cytokinesis execution programs. This incomplete reactivation pattern is consistent with the roles of our identified candidates: *ANLN* coordinates contractile ring assembly essential for cytokinesis (Engel et al. 2006), whereas *KIF18A* contributes to proper chromosome segregation (Mayr et al. 2007). Their insufficient transcriptional reactivation in adult IZ identifies specific molecular positions at which the cell‐cycle program stalls.

Comparative regulatory analysis revealed fundamental differences in transcriptional control between the developmental and adult contexts. Although both share core proliferation‐associated transcription factors (*CEBPB*, *FLI1*, *JUND*, and *MYB*), their downstream programs have diverged significantly. Developmental ccCM^+^ engage comprehensive cell‐cycle modules orchestrated by E2F family members (Ebelt et al. 2005) and chromatin remodelers, such as *SMARCA4* (Hang et al. 2010), creating a permissive environment for division. In contrast, adult ccCM^+^ preferentially activate stress‐adaptive regulons, including *BHLHE41* (Bret et al. 2025), *DDIT3* (Zhan et al. 2022), and *TEAD4* (Currey et al. 2021), reflecting an adaptation to hypoxia and proteotoxic stress rather than productive division. This shift from growth‐oriented to stress‐responsive transcriptional programs represents a cell‐autonomous barrier to regeneration (Sadek and Olson 2020).

The intercellular signaling landscape provides a critical context for understanding regenerative limitations. Developmental cardiomyocytes receive redundant proliferative inputs from multiple cellular sources, including the FGF (Khosravi et al. 2021), IGF (Li et al. 2011), NRG (Polizzotti et al. 2015), WNT (Lian et al. 2012), and BMP (Andree et al. 1998) pathways, creating a robust growth‐supportive environment. Adult myocardial infarction partially reactivates select pathways, particularly NRG, BMP, and IGF signaling, but superimposes injury‐specific signals, including SPP1 and PERIOSTIN, which favor fibrosis over proliferation (Kanisicak et al. 2016). This restricted and altered signaling environment fails to provide sufficient pro‐proliferative signaling. Together with the cell‐autonomous transcriptional barriers described above, these findings suggest that therapeutic strategies should address both intrinsic regulatory programs and microenvironmental signals. The dual constraint of cell‐autonomous barriers and non‐permissive microenvironmental signaling identified in our study parallels observations in other regenerative contexts. In the liver, inflammatory and fibrotic microenvironments suppress hepatocyte proliferation despite preserved regenerative capacity (Michalopoulos and Bhushan 2021). In skeletal muscle, the balance between pro‐regenerative and pro‐fibrotic immune signals determines satellite cell fate, with chronic injury environments favoring fibrosis over myogenesis (Wosczyna and Rando 2018). Similar principles have been observed in other disease contexts, where microenvironmental stress and immune remodeling shape cell fate decisions (Ji, Yang, et al. 2025; Yang et al. 2024). The cardiac context adds unique complexity through metabolic maturation, multinucleation, and sarcomeric organization, which impose additional cell‐intrinsic barriers beyond microenvironmental constraints. These cross‐organ parallels suggest that successful regenerative strategies must address both cellular competence and microenvironmental permissiveness.

Among the five candidates, the iPSC‐CM experiments revealed functionally distinct behaviors under conditions modeling the ischemic microenvironment. In immature cardiomyocytes under normoxia, CHIR99021 significantly enhanced *ANLN*, *KIF18A*, *MDK*, and *MKI67* expression, indicating that these genes retain Wnt responsiveness when the microenvironment is permissive. However, under hypoxic stress, this responsiveness was selectively preserved: *MDK* maintained significant CHIR‐mediated recovery, consistent with its cardioprotective role during ischemia–reperfusion injury (Horiba et al. 2006), representing a pharmacologically tractable candidate aligned with chemical biology approaches. *KIF18A* showed modest recovery, whereas *ANLN* remained unresponsive to CHIR stimulation under hypoxia, indicating that the ischemic microenvironment overrides Wnt‐mediated induction of late mitotic execution genes. Both *ANLN* and *KIF18A* are among the most strongly downregulated genes during postnatal cardiac maturation (bulk RNA‐seq log_2_FC > 5, FDR < 10^−28^), and their near‐absent baseline expression in mature Day 90 cardiomyocytes (relative expression < 0.2) suggests that developmental silencing constitutes an additional barrier to their reactivation in the adult context. *RTKN2* and *SOX11* exhibited a distinct pattern: both were suppressed rather than enhanced by Wnt activation under normoxia, and hypoxia abolished any further CHIR effect, suggesting that their re‐expression would require Wnt‐independent strategies. Collectively, these heterogeneous responses indicate that restoring complete cell‐cycle execution in adult cardiomyocytes will likely require combinatorial strategies addressing both the suppressive ischemic microenvironment and the developmental silencing of late mitotic execution genes.

Our findings help explain why adult hearts fail to regenerate despite the evidence of cell‐cycle activity. Previous studies have documented rare cardiomyocyte division events in adult hearts (Canseco et al. 2015; Mollova et al. 2013) and *MKI67* expression in peri‐infarct regions (Meckert et al. 2005; Vagnozzi et al. 2018); however, functional recovery remains negligible. Our analysis revealed that while proliferation markers are present in approximately 5% of the cardiomyocytes within the IZ, these cells show insufficient reactivation of execution factors associated with cytokinesis completion. Although this percentage may seem modest, the substantial cardiomyocyte loss in the IZ indicates that the successful regeneration of even this small proportion could meaningfully contribute to functional recovery. The hypoxic suppression of candidate gene expression demonstrated in our iPSC‐CM experiments suggests that the ischemic microenvironment may actively inhibit cell‐cycle program reactivation rather than merely failing to support it. This molecular‐level understanding indicates that therapeutic strategies should consider not only stimulating cell‐cycle entry but also enabling division completion through the restoration of execution machinery.

This study had several limitations that warrant consideration. First, although ccCM^+^ constitute approximately 5% of the cardiomyocyte population within IZ, extensive cardiomyocyte depletion in these regions results in relatively small absolute cell numbers for analysis. Secondly, transcriptional markers cannot definitively distinguish between true cytokinesis and endoreplication or binucleation events (Hesse et al. 2018). Future studies incorporating lineage tracing or live imaging are necessary to validate functional proliferation. Third, our computational analyses rely on algorithmic inference that should be interpreted within their respective methodological frameworks: AUCell captures gene signature enrichment rather than definitive cell states, hdWGCNA identifies co‐expression architecture rather than causal regulatory relationships, and CellChat infers potential signaling interactions from curated databases rather than measuring direct ligand‐receptor binding. Fourth, our candidate gene selection through multi‐omics integration, although rigorous, might have excluded other important regulators of cardiomyocyte cell‐cycle programs. Fifth, although our iPSC‐CM model provides human‐specific insights critical for translation, these cells exhibit incomplete maturation compared to adult cardiomyocytes (Tohyama et al. 2013), and our qPCR experiments assessed expression levels rather than functional proliferative capacity. Finally, spatial transcriptomics captures mixed signals from multiple cells per spot, limiting cell type‐specific attribution.

In conclusion, our integrative analysis of multiple public datasets revealed molecular features underlying incomplete cell‐cycle program reactivation in adult human infarcted hearts: partial reactivation of cell‐cycle machinery without proportional engagement of execution factors necessary for completing cell division, within an ischemic microenvironment that suppresses regenerative programs. The identification of specific developmental programs with limited adult reactivation provides candidate targets for future functional investigations. In particular, the maintenance of the responsiveness of *MDK* to pro‐proliferative signals under stress warrants further functional validation and mechanistic studies. These findings suggest that successful regenerative strategies may need to combine microenvironmental optimization with targeted restoration of execution factors. This human‐specific mechanistic understanding provides the foundation for future studies aimed at developing cardiac regenerative therapies.

## 4 Methods

### 4.1 Public Resources

We performed an integrative cross‐context analysis of human heart datasets spanning fetal and pediatric development and adult AMI. Public resources include snRNA‐seq, bulk RNA‐seq, bulk ATAC‐seq, and 10× Visium spatial transcriptomics. Data were obtained from public repositories (details and accession identifiers are provided in the Data Availability section and Tables S1 and S2). The reads and gene models were aligned to GRCh38 using GENCODE v38 (Frankish et al. 2019) annotations.

Cohorts were defined as follows. For developmental analysis, metadata classified samples at approximately 20 weeks post‐conception as fetal and at 4–14 years as pediatric, comprising snRNA‐seq (3 fetal, 3 pediatric samples), bulk RNA‐seq (6 fetal, 4 pediatric), and bulk ATAC‐seq (3 fetal, 4 pediatric). For adult analysis, AMI cases sampled within 12 days post‐infarction were included, and ischemic cardiomyopathy (ICM) and chronic heart failure (HF) samples were excluded. The adult myocardial tissue was annotated as IZ or RZ according to the source metadata. The adult cohort comprised snRNA‐seq (9 AMI, 4 controls) and spatial transcriptomics (13 AMI samples). A complete overview of the samples, modalities, groupings, and platforms is provided in Table S1 (development) and Table S2 (adult/AMI).

### 4.2 Ethics Statement

This study analyzes de‐identified and publicly available human datasets. No new human or animal participants were recruited for this study. Original datasets were collected with ethical approval and informed consent as follows: fetal/pediatric hearts from Royal Children's Hospital Melbourne (HREC 38192, 33001A, 37172A, 36358A), University of Queensland (2014000329), QIMR Berghofer (P2385), and University of Sydney (2016/923); adult hearts from Ruhr University Bochum (220–640), RWTH Aachen (EK151/09), University Medical Center Utrecht (12/387), and Washington University St. Louis (201104172). All the original studies complied with the principles of the Declaration of Helsinki. Complete ethical documentation is provided in Tables S1 and S2. iPSC‐cardiomyocyte experiments were performed using established cell lines without the recruitment of new subjects.

### 4.3 Bulk RNA‐Seq and ATAC‐Seq Processing

Bulk RNA‐seq count matrices were analyzed using DESeq2 (v1.46.0; Love et al. 2014). Genes with fewer than 10 counts across all samples were excluded. Size factors were calculated using the median ratio method. Differential expression was assessed using the Wald test implemented in DESeq2, with gene‐level dispersion estimates obtained through the default empirical Bayes procedure. Unless otherwise specified, significance required a Benjamini and Hochberg (1995) false discovery rate (FDR) < 0.05, and |log_2_ fold change| > 0.5.

Bulk ATAC‐seq data were provided as annotated peak‐by‐sample count matrices, and peak calling was not repeated. Peak‐level differential accessibility between fetal and pediatric samples was assessed using DESeq2 with the same multiple testing framework. Peaks detected in fewer than two samples per group were excluded prior to differential analysis. For downstream functional enrichment, the background gene set comprised all genes associated with peaks retained after this filtering step.

### 4.4 Single‐Nucleus RNA‐Seq Preprocessing and Integration

Developmental FASTQ files (10× Genomics Chromium) were processed using Cell Ranger (v9.0.1) against GRCh38 to produce genebarcode matrices. Adult snRNA‐seq objects were imported from 10× HDF5 files using Seurat (v5.2.1; Hao et al. 2021). Quality control retained nuclei with nFeature_RNA > 500 and percent mitochondrial reads < 15% and removed upper outliers based on sample‐specific distributions. Doublets were identified using DoubletFinder with the default parameters.

Normalization was performed using SCTransform (Hafemeister and Satija 2019) with 3000 variable features. Integration across donors was performed using Seurat's anchor‐based approach with the default parameters. Dimensionality reduction was performed using PCA, followed by UMAP (McInnes et al. 2018) (n.neighbors = 30, min.dist = 0.3). The clustering was performed at a resolution of 0.8.

### 4.5 Spatial Transcriptomics Processing and Validation

10× Visium data (8 IZ and 5 RZ) with preexisting cell‐type annotations from the original study (Kuppe et al. 2022) were imported using Seurat's Read10X_h5 function. Spots with < 500 UMIs or greater than 15% mitochondrial content were excluded. The data were normalized using SCTransform with 3000 variable features. For quantitative analyses, proliferation program scores per spot were calculated using AUCell, and the proportion of spots with detectable expression (raw UMI count > 0) was determined. Comparisons between the IZ and RZ were performed using two‐sided Wilcoxon rank‐sum tests with Benjamini–Hochberg correction.

### 4.6 Cardiomyocyte Subclustering and Cell‐Cycle‐Active State Classification

Cardiomyocytes were subsetted and re‐clustered at a resolution of 0.8 using the standard Seurat workflow. Cell‐cycle activity was quantified using AUCell (Aibar et al. 2017), a rank‐based scoring algorithm robust to library‐size variation and dropout events characteristic of sparse single‐nucleus data, with eight canonical proliferation markers (Mohamed et al. 2018; Nguyen et al. 2024; Whitfield et al. 2006): *AURKB*, *BUB1*, *CCNB1*, *CDCA8*, *CDK1*, *MKI67*, *PLK1*, and *TOP2A*. Cardiomyocytes exceeding the threshold were classified as ccCM^+^; the remaining were classified as ccCM^−^. This classification identifies cells with elevated cell‐cycle‐associated gene expression, which may encompass endomitosis and polyploidization events in addition to productive division (Meckert et al. 2005).

A unified threshold of 0.03 was applied across both datasets, selected from systematic evaluation of thresholds ranging from 0.01 to 0.10. In the developmental dataset, thresholds ≤ 0.02 yielded a higher ccCM^+^ proportion in pediatric than in fetal samples, contradicting the established postnatal decline in cardiomyocyte proliferative capacity (Mollova et al. 2013); 0.03 was the lowest value correctly recapitulating this hierarchy. In the adult dataset, the regional enrichment pattern (IZ > RZ > Control) remained stable across all tested thresholds (Figure S1B), and was further confirmed by threshold‐free comparison of per‐donor mean AUCell scores (Kruskal–Wallis, *p* = 0.021; Figure S1D). AUCell and UCell scores computed on the same eight‐gene signature showed strong sample‐level concordance in both datasets (Figure S1E,F).

### 4.7 Differential Expression Analyses

To avoid pseudoreplication, differential expression analysis between the ccCM^+^ and ccCM^−^ states employed pseudobulk aggregation (Squair et al. 2021). Raw counts were summed per donor and cell state and analyzed using DESeq2. Genes with a total count < 10 were filtered. Statistical significance required Benjamini–Hochberg FDR < 0.05, and |log_2_ fold change| > 0.5. Single‐nucleus Wilcoxon rank‐sum tests served as supplementary validation.

### 4.8 High‐Dimensional Weighted Gene Co‐Expression Network Analysis (hdWGCNA)

To identify coordinated transcriptional programs that may be missed by single‐gene differential expression approaches, co‐expression modules associated with the ccCM^+^ state were identified using hdWGCNA (Langfelder and Horvath 2008; Morabito et al. 2023) in cardiomyocyte subsets. Soft‐thresholding power was determined using the testSoftPowers function, with *β* = 8 selected as the minimum value achieving scale‐free topology (*R* ^2^ = 0.80). Signed networks were constructed using a bi‐weight mid‐correlation for robustness to outliers. Modules were detected by dynamic tree cutting (minModuleSize = 50, deepSplit = 2) and merged at mergeCutHeight = 0.25 based on eigengene similarity. Hub genes were defined as the intersection of module genes with differentially expressed genes in ccCM^+^.

Module activity was quantified using UCell scores (Andreatta and Carmona 2021) and module‐trait associations were calculated using eigengene‐trait correlations, with the cell‐cycle‐active state as the primary trait. Statistical significance was assessed using Pearson's correlation with Benjamini–Hochberg correction (FDR < 0.05). The modules were automatically numbered in order of decreasing size, following standard WGCNA conventions.

This broad definition was used for intermediate network characterization; a more stringent criterion (module membership > 0.7 and gene significance > 0.5) was applied during final candidate prioritization (see Section 4.12).

### 4.9 Functional Enrichment Analyses

Over‐representation analyses were performed using ClusterProfiler (v4.14.6) (Yu et al. 2012). Gene identifiers were converted to HGNC symbols and mapped to Entrez IDs using org.Hs.eg.db. Unmapped genes were excluded. The analyses used gene set size limits of 10–500, with *p*‐values adjusted using the Benjamini–Hochberg method. Redundant GO terms were removed using the simplify function in clusterProfiler with default parameters. Pathways with FDR < 0.05 were considered significant.

### 4.10 Regulatory Network Inference (SCENIC)

Gene regulatory networks were inferred separately for developmental and adult cardiomyocytes using SCENIC (v1.2.4; Van de Sande et al. 2020). Networks were constructed using the GRNBoost2 and refined using the human cisTarget database (v9). Regulon activity scores were calculated using AUCell and were z‐normalized. State‐associated regulons were defined as those with > 50 target genes, a z‐score difference > 0.5 between ccCM^+^ and ccCM^−^, and Benjamini‐Hochberg‐adjusted *p* < 0.05.

### 4.11 Cell–Cell Communication Analysis (CellChat)

Ligand‐receptor interactions were inferred using CellChat (v1.6.1; Jin et al. 2021, 2025), an approach that has been applied to characterize microenvironmental signaling in diverse disease‐context single‐nucleus studies (Ji, Jiang, et al. 2025; Yang et al. 2025). A minimum of 10 cells per group and a probability threshold of 0.05 were used. Statistical significance was assessed using 1000 permutations with Benjamini–Hochberg correction. For balanced comparisons, the larger groups were randomly sampled. The results were visualized as pathway heat maps (z‐normalized) and sender‐receiver networks, with ccCM^+^‐associated interactions highlighted.

### 4.12 Multi‐Omics Integration for Candidate Gene Prioritization

Evidence from multiple data modalities was integrated to identify developmental proliferation regulators with limited adult reactivation. Candidate genes were required to satisfy four developmental inclusion criteria and pass adult exclusion filters, following the Boolean logic:

$$\begin{array}{c} \text{Candidates}=\left(\mathrm{Dev}\_{\text{ccCM}}^{+}\_\mathrm{up}\cap \mathrm{Dev}\_\text{hdWGCNA}\_\mathrm{hub}\cap \right.\\ \left.\;,\text{Bulk},\_,\text{fetal},\_,\mathrm{up},\cap ,\text{Fetal},\_,\text{promoter},\_,\text{accessible}\right)\backslash \\ \;\left(\text{Adult}\_{\text{ccCM}}^{+}\_\mathrm{up}\cup \text{Adult}\_\text{hdWGCNA}\_\mathrm{hub}\right) \end{array}$$

where ∩ denotes set intersection, ∪ denotes set union, and \ denotes set difference (Figure 4C).

Developmental inclusion criteria were applied sequentially: (i) upregulation in developmental ccCM^+^ versus ccCM^−^ in snRNA‐seq (Benjamini–Hochberg FDR < 0.05; 832 genes); (ii) hub gene status within developmental hdWGCNA proliferation‐associated modules M3/M6 (module membership > 0.7, gene significance > 0.5; 279 genes retained); (iii) differential expression in fetal versus pediatric bulk RNA‐seq (FDR < 0.05, log_2_ fold change > 0.5; 184 genes retained); and (iv) increased chromatin accessibility at gene promoters in fetal versus pediatric ATAC‐seq (FDR < 0.05; 7 genes retained). Adult reactivation was then assessed: genes were excluded if upregulated in adult AMI ccCM^+^ versus ccCM^−^ (log_2_ fold change > 0.5) or identified as hub genes in AMI‐specific proliferation modules M5/M6; no genes were excluded at this step, indicating that all seven developmental candidates showed insufficient reactivation in the adult context. After curation excluding genes lacking protein‐coding annotation or established cardiac expression (*AC016205.1*, an unannotated lncRNA, and *NXPH2*, a neuron‐specific gene), five candidate genes remained: *ANLN*, *KIF18A*, *MDK*, *RTKN2*, and *SOX11*.

### 4.13 iPSC‐Cardiomyocyte Culture and Differentiation

Human iPSCs (line 201B7; RIKEN Bioresource Center, Tsukuba, Japan) were differentiated into cardiomyocytes using established small‐molecule Wnt modulation protocols (Lian et al. 2012). iPSCs were seeded at 1.6 × 10^5^ cells/cm^2^ on Geltrex‐coated surfaces (1:100 dilution) in StemFit AK02N medium (Ajinomoto) supplemented with 10 μM Y‐27632. When the cells reached 80%–90% confluence (typically on Day 0), differentiation was initiated by treatment with 9 μM CHIR99021 in RPMI‐1640 medium supplemented with B27 minus insulin for 24 h. On Day 1, the medium was replaced with fresh RPMI‐1640/B27 minus insulin. On Day 3, cells were treated with 5 μM IWP‐2 in the same basal medium for 48 h. From Day 5, the cells were maintained in RPMI‐1640/B27 minus insulin, and then switched to RPMI‐1640/B27 plus insulin from Day 7 onwards. Spontaneous beating was typically observed between Days 8 and 10. Metabolic purification was performed from Day 9 using glucose‐free RPMI supplemented with 5 mM lactate and B27 according to established protocols (Tohyama et al. 2013). Following purification, cells were dissociated using 0.25% trypsin–EDTA and re‐plated at 2.1 × 10^5^ cells/cm^2^ on Geltrex‐coated plates (1:100 dilution).

Experiments were conducted on Day 14 (immature) or Day 90 (mature). For maturation until Day 90, cells were maintained in RPMI‐1640/B27 with medium changes every 2–3 days. To model the ischemic microenvironment, cells were subjected to combined nutrient deprivation and hypoxia: serum‐ and glucose‐starved for 24 h in glucose‐free, serum‐free RPMI under normoxic conditions, followed by 12 h of hypoxic exposure (1% O_2_, 5% CO_2_, balance N_2_) in the same medium. Normoxic controls were maintained in standard RPMI‐1640/B27 medium under standard atmospheric conditions (21% O_2_, 5% CO_2_) for the equivalent duration. For Wnt activation in Day 14 experiments, 2 μM CHIR99021 was added at the onset of starvation and maintained throughout hypoxic exposure.

### 4.14 Quantitative PCR Validation

Total RNA was isolated immediately after the treatment using standard RNA extraction protocols. qPCR was performed using the QuantStudio 7 platform (Applied Biosystems) with SYBR Green qPCR Master Mix and custom primers (sequences provided in Table S3). Target genes included *BNIP3* and *SLC2A1* as hypoxia response markers (Semenza 2014), *MKI67* as a proliferation marker, and the candidate genes *ANLN*, *KIF18A*, *MDK*, *RTKN2*, and *SOX11*. Cycle threshold values were normalized to GAPDH and analyzed using the 2^−^ΔΔCt method (Livak and Schmittgen 2001).

The experiments included eight biological replicates with at least two technical replicates per condition. Two‐factor designs were analyzed using two‐way ANOVA with Tukey's honest significant difference test for post hoc comparisons (Tukey 1949).

### 4.15 Statistical Analyses

Statistical analyses were performed using R (v4.4.1). All tests were two‐sided, and significance was defined as an adjusted *p* < 0.05. For single‐nucleus data, pseudo‐bulk DESeq2 served as the primary analysis. Multiple comparisons were corrected using the Benjamini–Hochberg method. Two‐factor experiments were performed using a two‐way ANOVA with Tukey's post hoc test. The sample sizes and adjusted *p*‐values are provided in the figure legends.

## Author Contributions

**Jun Li:** conceptualization, methodology, validation, writing – original draft, writing – review and editing. **Kyoko Hidaka:** conceptualization, methodology, investigation, funding acquisition, writing – review and editing. **Jieran Lyu:** conceptualization, methodology, investigation, validation, formal analysis, visualization, writing – original draft. **Jong‐Kook Lee:** conceptualization, methodology, project administration, resources, writing – review and editing, funding acquisition. **Yuki Kuramoto:** methodology, writing – review and editing. **Yasushi Sakata:** writing – review and editing, supervision.

## Funding

This work was supported by the Japan Society for the Promotion of Science (JSPS) KAKENHI [JP22K11733].

## Conflicts of Interest

The authors declare no conflicts of interest.

## Supporting information


**Figure S1:** AUCell‐based classification of cell‐cycle‐active cardiomyocytes and threshold validation. (A) UMAP visualization of AUCell scores in adult AMI (top) and developmental (bottom) cardiomyocytes. (B) Threshold sensitivity analysis showing ccCM^+^ proportion across AUCell thresholds (0.01–0.10) in adult (top) and developmental (bottom) datasets. Dashed line indicates the selected threshold of 0.03 (see Section 4 for selection rationale). (C) Distribution of AUCell scores in adult (top) and developmental (bottom) cardiomyocytes. (D) Threshold‐free comparison of per‐donor mean AUCell scores across adult cardiac regions (*n* = 4 control, *n* = 5 RZ, *n* = 4 IZ). Kruskal–Wallis *p* = 0.021. (E, F) Sample‐level concordance between AUCell and UCell scores computed on the same eight‐gene signature in adult (E) and developmental (F) datasets. Each point represents the mean score per sample; Spearman ρ and *p* values are indicated.
**Figure S2:** Characterization of cell‐cycle‐active cardiomyocytes in adult AMI samples. (A) UMAP visualization of major cardiac cell types from control, RZ, and IZ samples. (B) UMAP of cardiomyocytes with ccCM^+^ and ccCM^−^ classification indicated. (C) Feature density maps showing expression of the eight proliferation markers used for AUCell scoring across adult cardiomyocytes. (D) Dot plot of the eight proliferation markers across cell‐cycle states and regions. Dot size indicates percentage of expressing cells; color intensity represents average expression level. (E) Violin plots comparing S‐phase and G2/M‐phase scores between ccCM^+^ and ccCM^−^. Wilcoxon rank‐sum test; significance levels indicated. (F) GO Biological process enrichment of ccCM^+^ differentially expressed genes.
**Figure S3:** hdWGCNA network construction and module characterization in adult AMI cardiomyocytes. (A) Soft‐threshold power selection showing scale‐free topology model fit (*R* ^2^), mean, median, and max connectivity as a function of soft power. Dashed line indicates the selected *β* = 8 (*R* ^2^ = 0.80). (B) Hierarchical clustering dendrogram with 16 modules identified by dynamic tree cutting (color bars). (C) Module‐trait correlation heatmap. (D) UMAP overlays of module activity scores (UCell) across cardiomyocytes. Modules M5 and M6 show enrichment in the ccCM^+^ region. (E) Representative hub gene subnetworks for modules M5 and M6. Node size reflects module membership; edge thickness indicates connection strength. (F) GO and KEGG enrichment of combined M5/M6 gene sets.
**Figure S4:** Multi‐omics analysis of developmental cardiomyocyte proliferation. (A) UMAP visualization of snRNA‐seq data from fetal and pediatric hearts, colored by cell type. (B) Feature density maps of the eight proliferation markers used for AUCell scoring across developmental cardiomyocytes. (C) Volcano plots of fetal versus pediatric differential analysis from bulk RNA‐seq (top; 8646 DEGs) and bulk ATAC‐seq (bottom; 371 differential accessibility regions). Fetal‐upregulated features are highlighted. (D) GO Biological Process enrichment of the developmental cross‐validated gene set (defined in Figure 3E).
**Figure S5:** hdWGCNA network construction and module characterization in developmental cardiomyocytes. (A) Soft‐threshold power selection showing scale‐free topology model fit (*R* ^2^), mean, median, and max connectivity. Dashed line indicates the selected *β* = 8 (*R* ^2^ = 0.80). (B) Hierarchical clustering dendrogram with 11 modules identified by dynamic tree cutting (color bars). (C) Module‐trait correlation heatmap. (D) UMAP overlays of module activity scores (UCell) across developmental cardiomyocytes. Modules M3 and M6 show enrichment in fetal ccCM^+^ subclusters. (E) Representative hub gene subnetworks for modules M3 and M6. Node size reflects module membership; edge thickness indicates connection strength.
**Figure S6:** SCENIC‐inferred transcriptional regulatory networks in developmental and adult cardiomyocytes. (A) Network visualization integrating the five candidate genes with SCENIC‐identified transcription factors and enriched pathways in developmental hearts (see inset legend for node types). (B) Transcription factor (TF)–regulon networks in adult hearts. Upper panel: TFs with elevated activity in ccCM^+^; lower panel: TFs elevated in ccCM^−^. (C) TF–regulon networks in developmental hearts. Left: ccCM^+^‐associated TFs; right: ccCM^−^‐associated TFs. (D) UMAP overlay of FLI1 regulon activity in adult (upper) and developmental (lower) datasets.
**Figure S7:** CellChat analysis of intercellular communication networks across developmental and disease contexts. (A) Pathway‐level incoming signaling strength to each cell type in fetal (left) and pediatric (right) hearts. (B) Ligand–receptor interactions from indicated sender cell types to ccCM^+^ in the fetal cohort. Point color encodes communication probability; point size reflects significance. (C) Pathway‐level incoming signaling strength in adult IZ (left) and RZ (right). (D) Ligand–receptor interactions from indicated sender cell types to ccCM^+^ in adult AMI.
**Figure S8:** Spatial transcriptomic validation of cellular composition in myocardial infarction tissue. (A) Workflow for spatial transcriptomics data processing (details indicated in the flowchart). (B) UMAP visualization of spatial transcriptomics data, split by RZ and IZ, colored by cell type. (C) Cell‐type composition comparing IZ and RZ.
**Figure S9:** Validation of the ischemic–hypoxic exposure model in iPSC‐derived cardiomyocytes. qPCR analysis of BNIP3 and SLC2A1, canonical HIF‐1 target genes, confirming effective hypoxic exposure. Data represent mean ± SEM (*n* = 8 biological replicates). Two‐way ANOVA with Tukey's post hoc test; significance symbols are defined in the panel.

**Table S1:** Developmental dataset information. Summary of human developmental heart samples used in this study, including GEO accession numbers, sample identifiers, developmental stage, age, sex, assay type, cardiac region, and clinical diagnosis. Fetal samples range from 14 to 20 post‐conceptional weeks; pediatric samples range from 3 weeks to 14 years. All samples were obtained from left ventricular tissue of individuals without cardiovascular disease. Data were obtained from a previously published cohort (Sim et al. 2021).

**Table S2:** Adult AMI dataset information. Clinical and pathological characteristics of adult AMI and control samples analyzed in this study. Spatial transcriptomics samples (10× and ACH prefixes) and snRNA‐seq samples are listed with tissue zone, clinical metadata, and pathology descriptions from the original study. Data were obtained from a previously published cohort (Kuppe et al. 2022).

**Table S3:** qPCR primer sequences. Forward and reverse primers used for qPCR experiments in human iPSC‐derived cardiomyocytes. Primer pairs were obtained from PrimerBank (Wang et al. 2012) and were selected to span exon–exon junctions where possible to avoid genomic DNA amplification.

## Data Availability

Developmental datasets were obtained from Gene Expression Omnibus (GEO; GSE156703, GSE156702, and GSE156704; Sim et al. 2021). Adult AMI multi‐omics datasets (snRNA‐seq and spatial data) were retrieved from Zenodo (6578617 and 6580069; Kuppe et al. 2022). Additional processed matrices and metadata generated in this study are available from the corresponding author upon reasonable request. Code availability: The custom‐analysis scripts and detailed parameter settings are available from the corresponding author upon request.
