# Supplementary material for: Multi‐Omics Integration Reveals Incomplete Reactivation of Developmental Cell‐Cycle Programs in Adult Human Infarcted Hearts
Source: Chem Biol Drug Des. 2026 Jun 8;107(6):e70337. doi: 10.1111/cbdd.70337 (PMC13247538; doi:10.1111/cbdd.70337)
Supplement: Supplementary file 1 — Figure S1: AUCell‐based classification of cell‐cycle‐active cardiomyocytes and threshold validation. (A) UMAP visualization of AUCell scores in adult AMI (top) and developmental (bottom) cardiomyocytes. (B) Threshold sensitivity analysis showing ccCM+ proportion across AUCell thresholds (0.01–0.10) in adult (top) and developmental (bottom) datasets. Dashed line indicates the selected threshold of 0.03 (see Section 4 for selection rationale). (C) Distribution of AUCell scores in adult (top) and developmental (bottom) cardiomyocytes. (D) Threshold‐free comparison of per‐donor mean AUCell scores across adult cardiac regions (n = 4 control, n = 5 RZ, n = 4 IZ). Kruskal–Wallis p = 0.021. (E, F) Sample‐level concordance between AUCell and UCell scores computed on the same eight‐gene signature in adult (E) and developmental (F) datasets. Each point represents the mean score per sample; Spearman ρ and p values are indicated. Figure S2: Characterization of cell‐cycle‐active cardiomyocytes in adult AMI samples. (A) UMAP visualization of major cardiac cell types from control, RZ, and IZ samples. (B) UMAP of cardiomyocytes with ccCM+ and ccCM− classification indicated. (C) Feature density maps showing expression of the eight proliferation markers used for AUCell scoring across adult cardiomyocytes. (D) Dot plot of the eight proliferation markers across cell‐cycle states and regions. Dot size indicates percentage of expressing cells; color intensity represents average expression level. (E) Violin plots comparing S‐phase and G2/M‐phase scores between ccCM+ and ccCM−. Wilcoxon rank‐sum test; significance levels indicated. (F) GO Biological process enrichment of ccCM+ differentially expressed genes. Figure S3: hdWGCNA network construction and module characterization in adult AMI cardiomyocytes. (A) Soft‐threshold power selection showing scale‐free topology model fit (R 2), mean, median, and max connectivity as a function of soft power. Dashed line indicates the selected β = 8 (R 2 [file CBDD-107-e70337-s003.pdf]

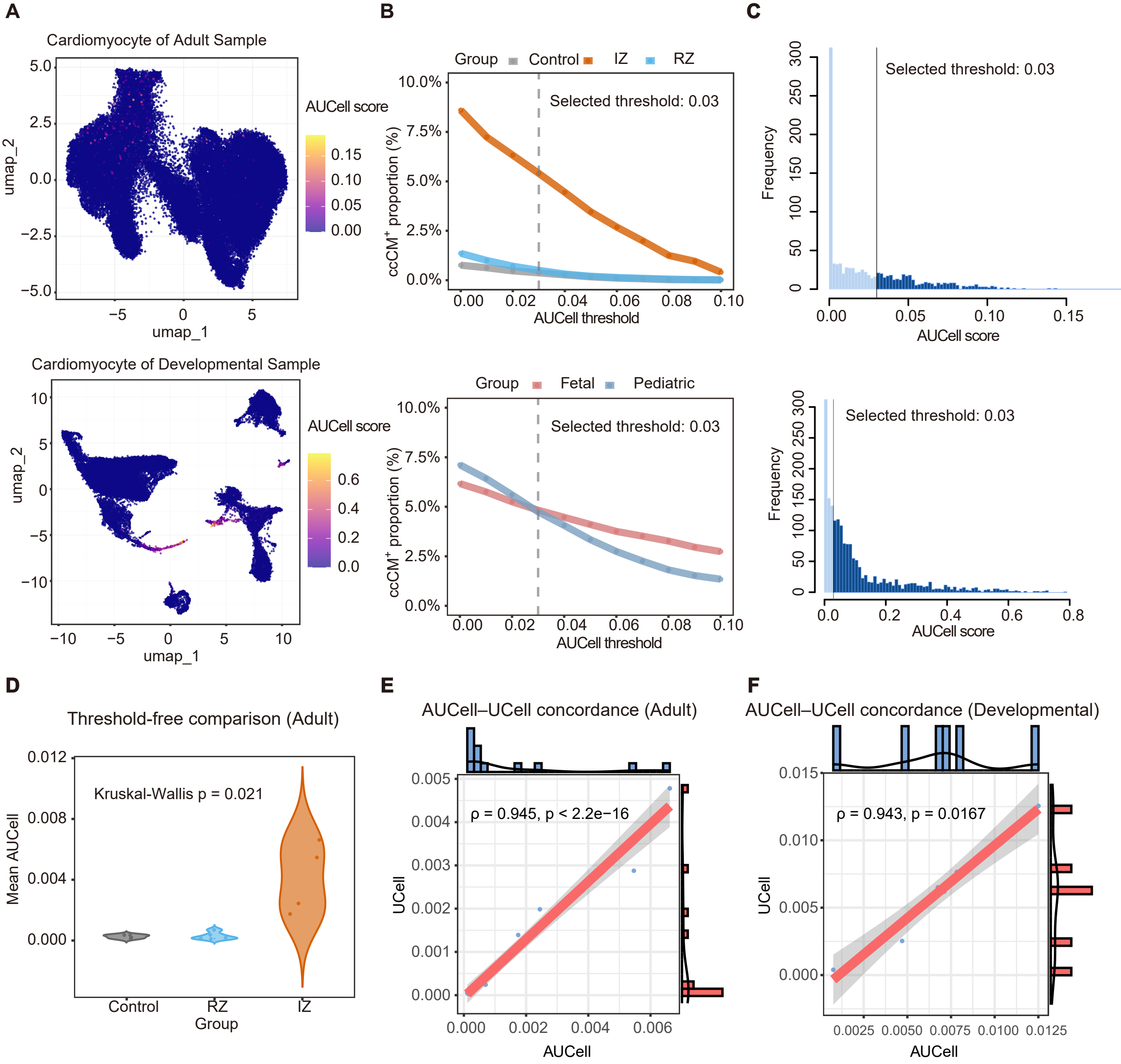
A
Cardiomyocyte of Adult Sample
AUCell score
umap_1
umap_2
Cardiomyocyte of Developmental Sample
B
Group Control IZ RZ
Selected threshold: 0.03
ccCM+ proportion (%)
AUCell threshold
Group Fetal Pediatric
C
Selected threshold: 0.03
Frequency
AUCell score
D
Threshold-free comparison (Adult)
Kruskal-Wallis p = 0.021
Mean AUCell
Control
RZ
IZ
Group
E
AUCell–UCell concordance (Adult)
ρ = 0.945, p < 2.2e−16
UCell
AUCell
F
AUCell–UCell concordance (Developmental)
ρ = 0.943, p = 0.0167

Supplementary Fig. S1 AUCell-based classification of cell-cycle-active cardiomyocytes and threshold validation. (A) UMAP visualization of AUCell scores in adult AMI (top) and developmental (bottom) cardiomyocytes. (B) Threshold sensitivity analysis showing $ccCM^+$ proportion across AUCell thresholds (0.01–0.10) in adult (top) and developmental (bottom) datasets. Dashed line indicates the selected threshold of 0.03 (see Methods for selection rationale). (C) Distribution of AUCell scores in adult (top) and developmental (bottom) cardiomyocytes. (D) Threshold-free comparison of per-donor mean AUCell scores across adult cardiac regions (n = 4 control, n = 5 RZ, n = 4 IZ). Kruskal–Wallis P = 0.021. (E–F) Sample-level concordance between AUCell and UCell scores computed on the same eight-gene signature in adult (E) and developmental (F) datasets. Each point represents the mean score per sample; Spearman ρ and P values are indicated.

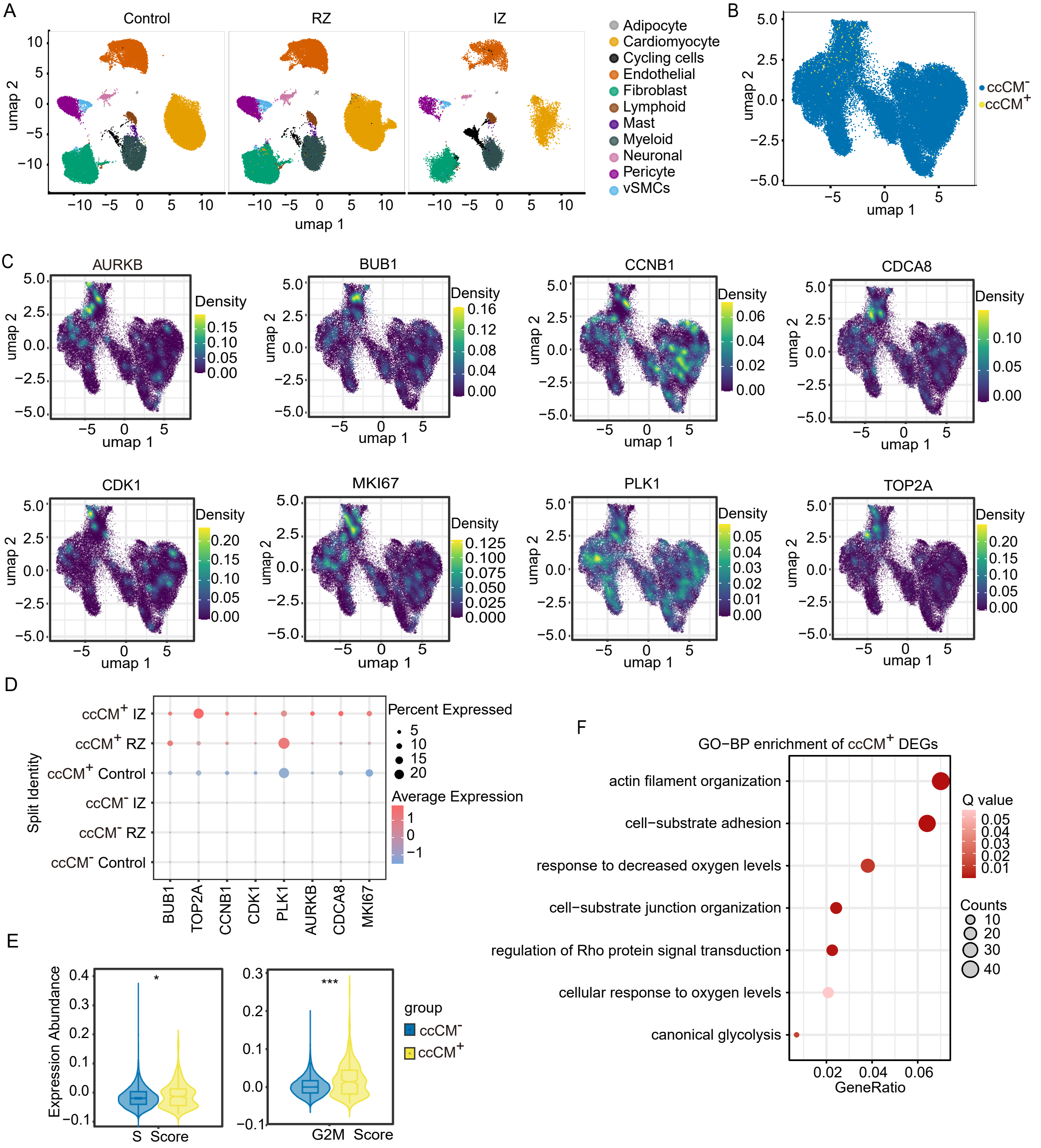
A
Control
RZ
IZ
umap 2
umap 1
Adipocyte
Cardiomyocyte
Cycling cells
Endothelial
Fibroblast
Lymphoid
Mast
Myeloid
Neuronal
Pericyte
vSMCs
B
umap 2
umap 1
ccCM-
ccCM+
C
AURKB
BUB1
CCNB1
CDCA8
CDK1
MKI67
PLK1
TOP2A
Density
umap 2
umap 1
D
ccCM+ IZ
ccCM+ RZ
ccCM+ Control
ccCM- IZ
ccCM- RZ
ccCM- Control
Split Identity
BUB1
TOP2A
CCNB1
CDK1
PLK1
AURKB
CDCA8
MKI67
Percent Expressed
Average Expression
E
Expression Abundance
S Score
G2M Score
group
ccCM-
ccCM+
F
GO−BP enrichment of ccCM+ DEGs
actin filament organization
cell−substrate adhesion
response to decreased oxygen levels
cell−substrate junction organization
regulation of Rho protein signal transduction
cellular response to oxygen levels
canonical glycolysis
Q value
Counts
GeneRatio

Supplementary Fig. S2 Characterization of cell-cycle-active cardiomyocytes in adult AMI samples. (A) UMAP visualization of major cardiac cell types from control, RZ, and IZ samples. (B) UMAP of cardiomyocytes with $ccCM^+$ and $ccCM^-$ classification indicated. (C) Feature density maps showing expression of the eight proliferation markers used for AUCell scoring across adult cardiomyocytes. (D) Dot plot of the eight proliferation markers across cell-cycle states and regions. Dot size indicates percentage of expressing cells; color intensity represents average expression level. (E) Violin plots comparing S-phase and G2/M-phase scores between $ccCM^+$ and $ccCM^-$. Wilcoxon rank-sum test; significance levels indicated. (F) GO Biological Process enrichment of $ccCM^+$ differentially expressed genes.

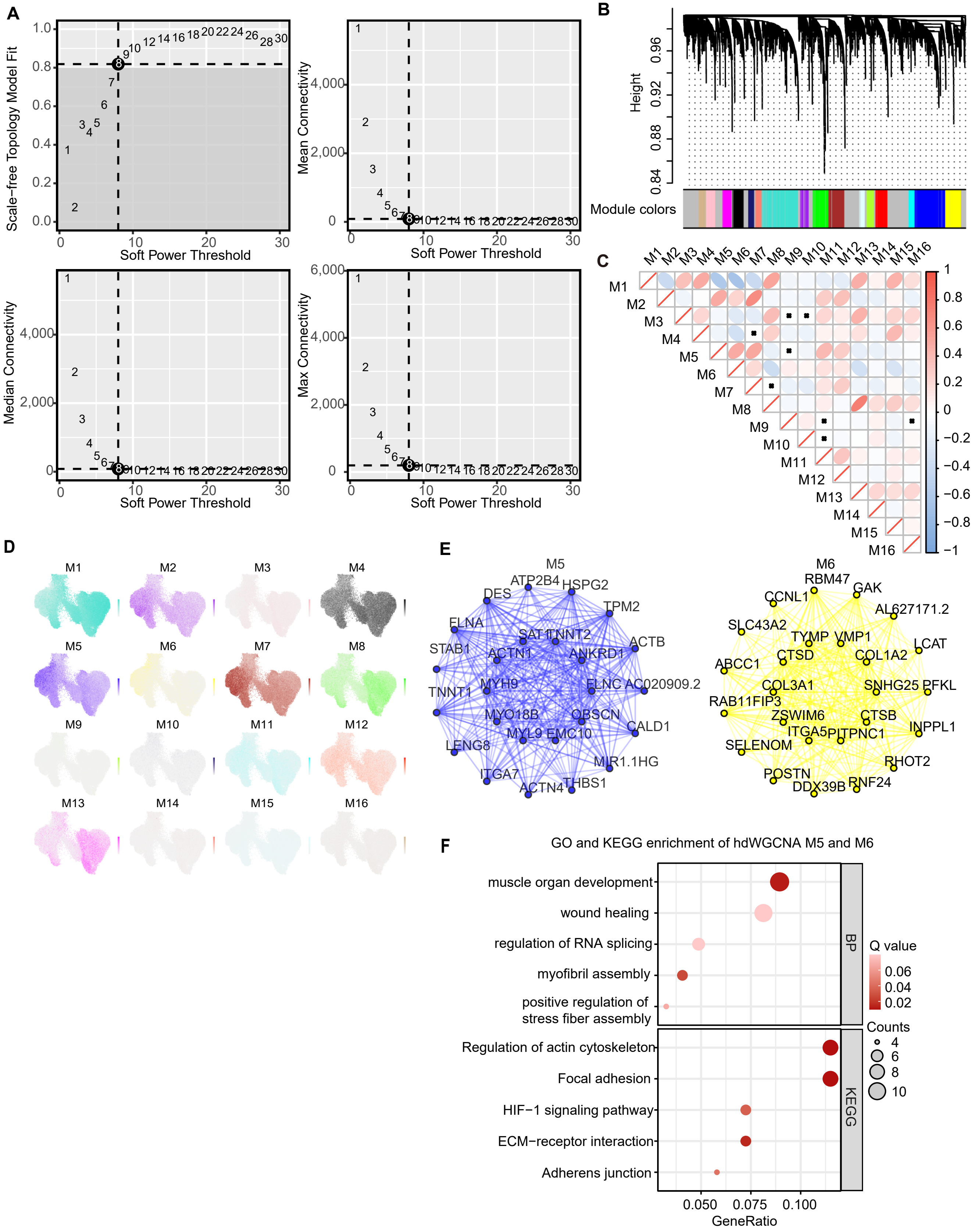

A
Scale−free Topology Model Fit
Soft Power Threshold
Mean Connectivity
Soft Power Threshold
Median Connectivity
Soft Power Threshold
Max Connectivity
Soft Power Threshold
B
Height
Module colors
C
M1 M2 M3 M4 M5 M6 M7 M8 M9 M10 M11 M12 M13 M14 M15 M16
D
M1 M2 M3 M4 M5 M6 M7 M8 M9 M10 M11 M12 M13 M14 M15 M16
E
M5
ATP2B4 HSPG2 DES TPM2 FLNA SAT TNNT2 ACTB STAB1 ACTN1 ANKRD1 TNNT1 MYH9 FLNC AC020909.2 MYO18B OBSCN CALD1 MYL9 EMC10 LENG8 MIR1.1HG ITGA7 ACTN4 THBS1
M6
RBM47 GAK CCNL1 AL627171.2 SLC43A2 TYMP VMP1 LCAT ABCC1 CTSD COL1A2 COL3A1 SNHG25 PFKL RAB11FIP3 ZSWIM6 CTSB INPPL1 ITGA5 PITPNC1 SELENOM RHOT2 POSTN RNF24 DDX39B
F
GO and KEGG enrichment of hdWGCNA M5 and M6
muscle organ development
wound healing
regulation of RNA splicing
myofibril assembly
positive regulation of stress fiber assembly
Regulation of actin cytoskeleton
Focal adhesion
HIF−1 signaling pathway
ECM−receptor interaction
Adherens junction
BP
KEGG
Q value
Counts
GeneRatio

Supplementary Fig. S3 hdWGCNA network construction and module characterization in adult AMI cardiomyocytes. (A) Soft-threshold power selection showing scale-free topology model fit (R^2), mean, median, and max connectivity as a function of soft power. Dashed line indicates the selected $\beta = 8$ ($R^2 = 0.80$). (B) Hierarchical clustering dendrogram with 16 modules identified by dynamic tree cutting (color bars). (C) Module-trait correlation heatmap. (D) UMAP overlays of module activity scores (UCell) across cardiomyocytes. Modules M5 and M6 show enrichment in the $ccCM^+$ region. (E) Representative hub gene subnetworks for modules M5 and M6. Node size reflects module membership; edge thickness indicates connection strength. (F) GO and KEGG enrichment of combined M5/M6 gene sets.

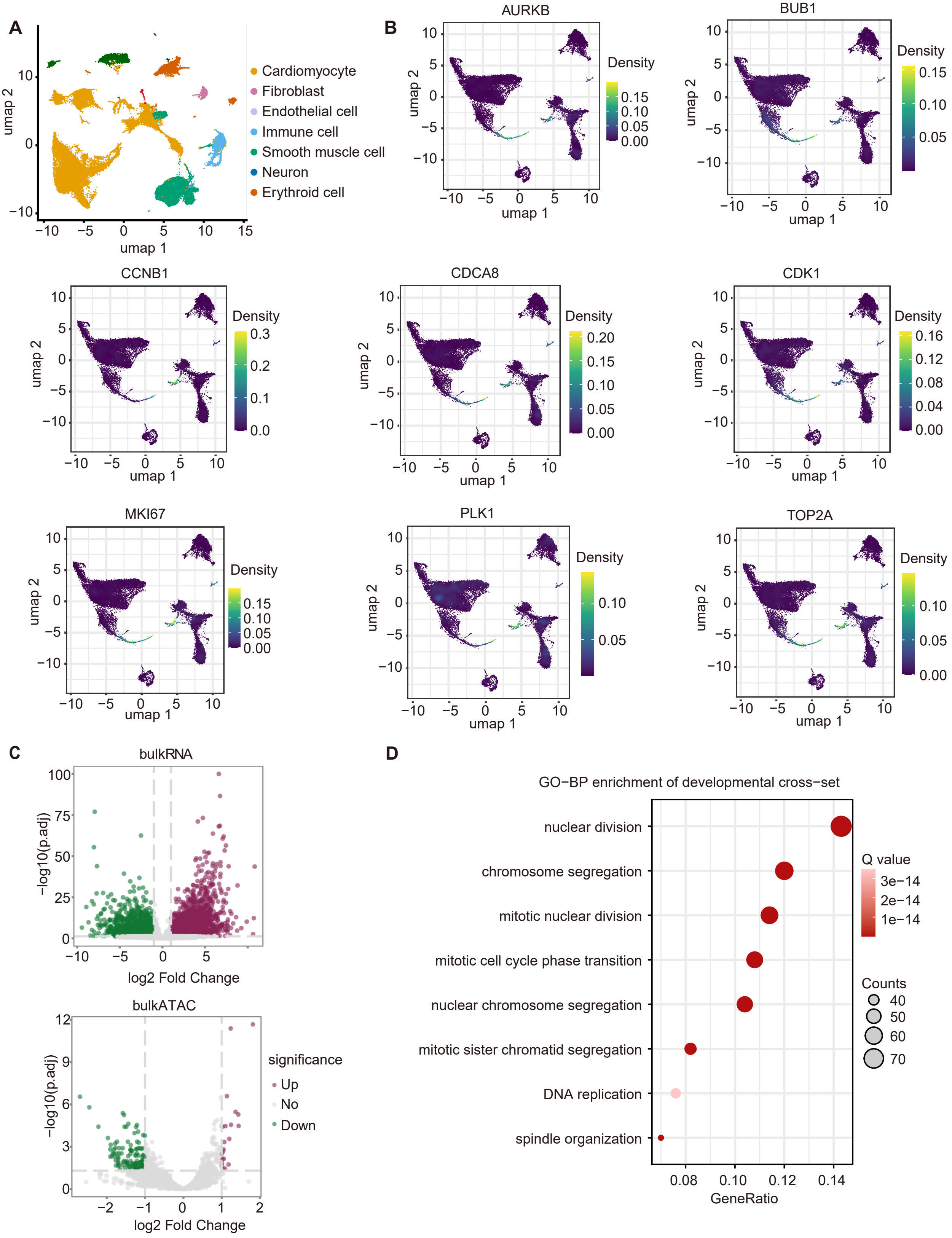
A
Cardiomyocyte
Fibroblast
Endothelial cell
Immune cell
Smooth muscle cell
Neuron
Erythroid cell
umap 1
umap 2
B
AURKB
BUB1
CCNB1
CDCA8
CDK1
MKI67
PLK1
TOP2A
Density
C
bulkRNA
bulkATAC
log2 Fold Change
−log10(p.adj)
significance
Up
No
Down
D
GO−BP enrichment of developmental cross−set
nuclear division
chromosome segregation
mitotic nuclear division
mitotic cell cycle phase transition
nuclear chromosome segregation
mitotic sister chromatid segregation
DNA replication
spindle organization
GeneRatio
Q value
Counts

Supplementary Fig. S4 Multi-omics analysis of developmental cardiomyocyte proliferation. (A) UMAP visualization of snRNA-seq data from fetal and pediatric hearts, colored by cell type. (B) Feature density maps of the eight proliferation markers used for AUCell scoring across developmental cardiomyocytes. (C) Volcano plots of fetal versus pediatric differential analysis from bulk RNA-seq (top; 8,646 DEGs) and bulk ATAC-seq (bottom; 371 differential accessibility regions). Fetal-upregulated features are highlighted. (D) GO Biological Process enrichment of the developmental cross-validated gene set (defined in Fig. 3E).

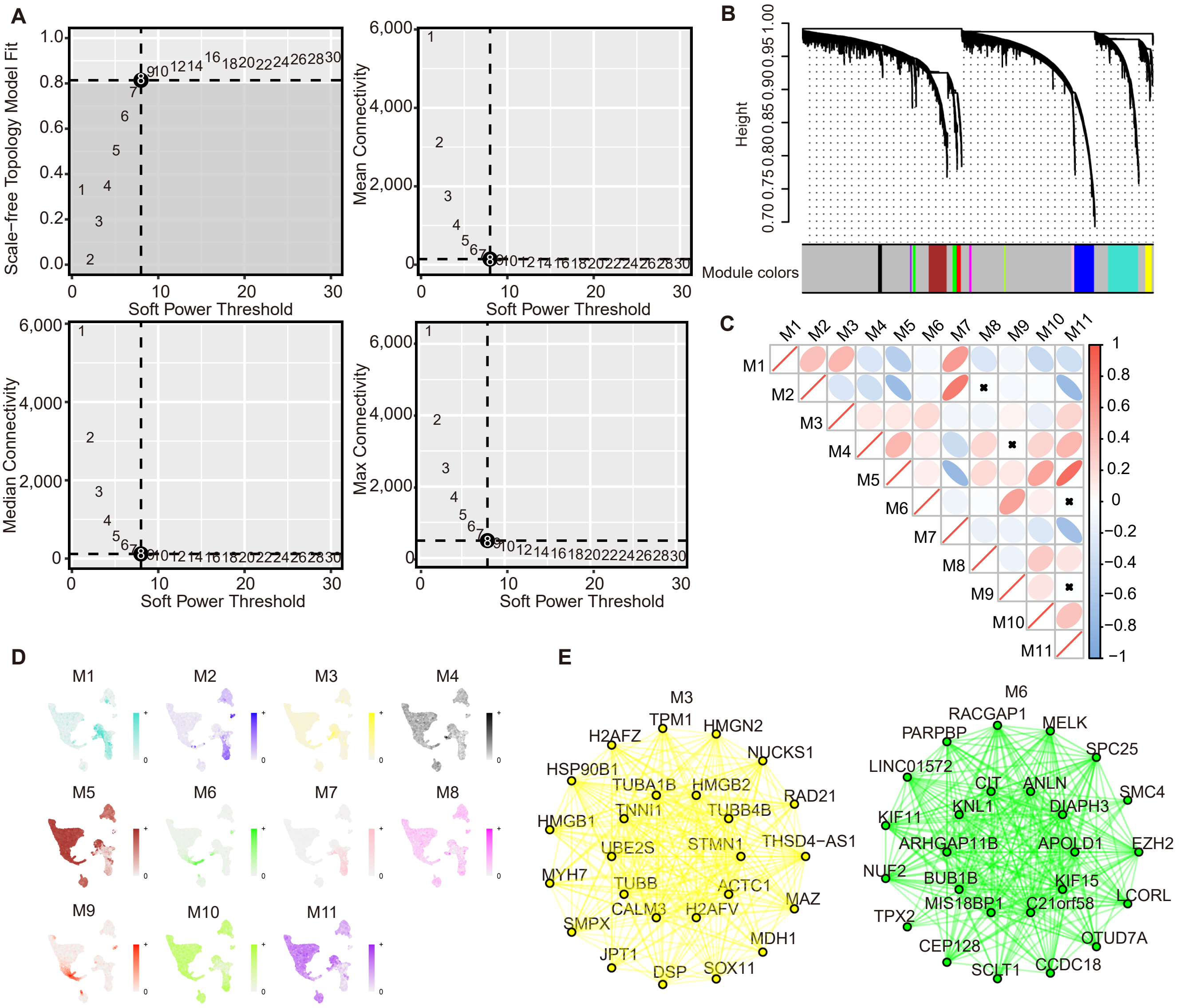

A
Scale-free Topology Model Fit
Mean Connectivity
Median Connectivity
Max Connectivity
Soft Power Threshold
B
Height
Module colors
C
M1 M2 M3 M4 M5 M6 M7 M8 M9 M10 M11
D
M1 M2 M3 M4 M5 M6 M7 M8 M9 M10 M11
E
M3
TPM1 HMGN2 H2AFZ NUCKS1 HSP90B1 TUBA1B HMGB2 RAD21 TNNI1 TUBB4B HMGB1 THSD4−AS1 UBE2S STMN1 MYH7 TUBB ACTC1 MAZ CALM3 H2AFV SMPX MDH1 JPT1 DSP SOX11
M6
RACGAP1 MELK PARPBP SPC25 LINC01572 CIT ANLN SMC4 KIF11 KNL1 DIAPH3 ARHGAP11B APOLD1 EZH2 NUF2 BUB1B KIF15 LCORL MIS18BP1 C21orf58 TPX2 OTUD7A CEP128 SCLT1 CCDC18

Supplementary Fig. S5 hdWGCNA network construction and module characterization in developmental cardiomyocytes. (A) Soft-threshold power selection showing scale-free topology model fit (R^2), mean, median, and max connectivity. Dashed line indicates the selected $\beta = 8$ ($R^2 = 0.80$). (B) Hierarchical clustering dendrogram with 11 modules identified by dynamic tree cutting (color bars). (C) Module-trait correlation heatmap. (D) UMAP overlays of module activity scores (UCell) across developmental cardiomyocytes. Modules M3 and M6 show enrichment in fetal $ccCM^+$ subclusters. (E) Representative hub gene subnetworks for modules M3 and M6. Node size reflects module membership; edge thickness indicates connection strength.

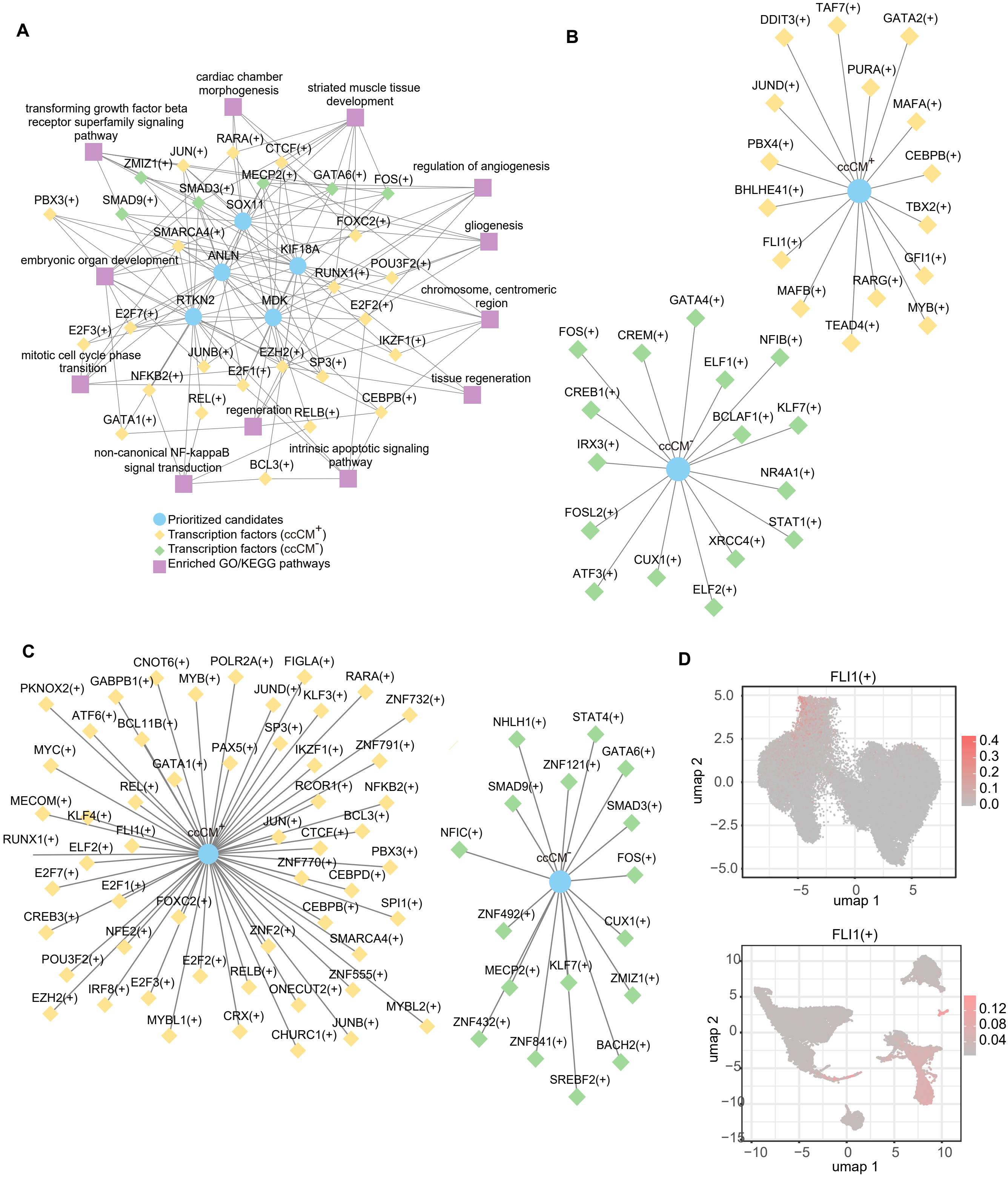
A
cardiac chamber morphogenesis
striated muscle tissue development
transforming growth factor beta receptor superfamily signaling pathway
regulation of angiogenesis
gliogenesis
embryonic organ development
chromosome, centromeric region
mitotic cell cycle phase transition
tissue regeneration
regeneration
non-canonical NF-kappaB signal transduction
intrinsic apoptotic signaling pathway
RARA(+)
CTCF(+)
JUN(+)
ZMIZ1(+)
MECP2(+)
GATA6(+)
FOS(+)
SMAD3(+)
PBX3(+)
SMAD9(+)
SOX11
FOXC2(+)
SMARCA4(+)
ANLN
KIF18A
POU3F2(+)
RUNX1(+)
RTKN2
MDK
E2F7(+)
E2F2(+)
E2F3(+)
IKZF1(+)
JUNB(+)
EZH2(+)
SP3(+)
NFKB2(+)
E2F1(+)
REL(+)
RELB(+)
CEBPB(+)
GATA1(+)
BCL3(+)
Prioritized candidates
Transcription factors (ccCM+)
Transcription factors (ccCM-)
Enriched GO/KEGG pathways
B
DDIT3(+)
TAF7(+)
GATA2(+)
PURA(+)
JUND(+)
MAFA(+)
PBX4(+)
CEBPB(+)
ccCM+
BHLHE41(+)
TBX2(+)
FLI1(+)
GFI1(+)
RARG(+)
MAFB(+)
MYB(+)
TEAD4(+)
GATA4(+)
FOS(+)
CREM(+)
NFIB(+)
ELF1(+)
CREB1(+)
KLF7(+)
BCLAF1(+)
IRX3(+)
ccCM-
NR4A1(+)
FOSL2(+)
STAT1(+)
XRCC4(+)
CUX1(+)
ATF3(+)
ELF2(+)
C
CNOT6(+)
POLR2A(+)
FIGLA(+)
RARA(+)
PKNOX2(+)
GABPB1(+)
MYB(+)
JUND(+)
KLF3(+)
ZNF732(+)
ATF6(+)
BCL11B(+)
SP3(+)
MYC(+)
PAX5(+)
IKZF1(+)
ZNF791(+)
GATA1(+)
REL(+)
RCOR1(+)
NFKB2(+)
MECOM(+)
KLF4(+)
JUN(+)
BCL3(+)
FLI1(+)
ccCM+
CTCF(+)
RUNX1(+)
ELF2(+)
PBX3(+)
ZNF770(+)
E2F7(+)
CEBPD(+)
E2F1(+)
SPI1(+)
FOXC2(+)
CREB3(+)
CEBPB(+)
NFE2(+)
ZNF2(+)
SMARCA4(+)
POU3F2(+)
E2F2(+)
ZNF555(+)
RELB(+)
E2F3(+)
IRF8(+)
ONECUT2(+)
EZH2(+)
MYBL2(+)
JUNB(+)
MYBL1(+)
CRX(+)
CHURC1(+)
NHLH1(+)
STAT4(+)
GATA6(+)
ZNF121(+)
SMAD9(+)
SMAD3(+)
NFIC(+)
ccCM-
FOS(+)
ZNF492(+)
CUX1(+)
MECP2(+)
KLF7(+)
ZMIZ1(+)
ZNF432(+)
ZNF841(+)
BACH2(+)
SREBF2(+)
D
FLI1(+)
5.0
2.5
0.0
−2.5
−5.0
umap 2
−5
0
5
umap 1
0.4
0.3
0.2
0.1
0.0
FLI1(+)
10
5
0
−5
−10
−15
umap 2
−10
−5
0
5
10
umap 1
0.12
0.08
0.04

Supplementary Fig. S6 SCENIC-inferred transcriptional regulatory networks in developmental and adult cardiomyocytes. (A) Network visualization integrating the five candidate genes with SCENIC-identified transcription factors and enriched pathways in developmental hearts (see inset legend for node types). (B) Transcription factor (TF)–regulon networks in adult hearts. Upper panel: TFs with elevated activity in $ccCM^+$; lower panel: TFs elevated in $ccCM^-$. (C) TF–regulon networks in developmental hearts. Left: $ccCM^+$-associated TFs; right: $ccCM^-$-associated TFs. (D) UMAP overlay of FLI1 regulon activity in adult (upper) and developmental (lower) datasets.

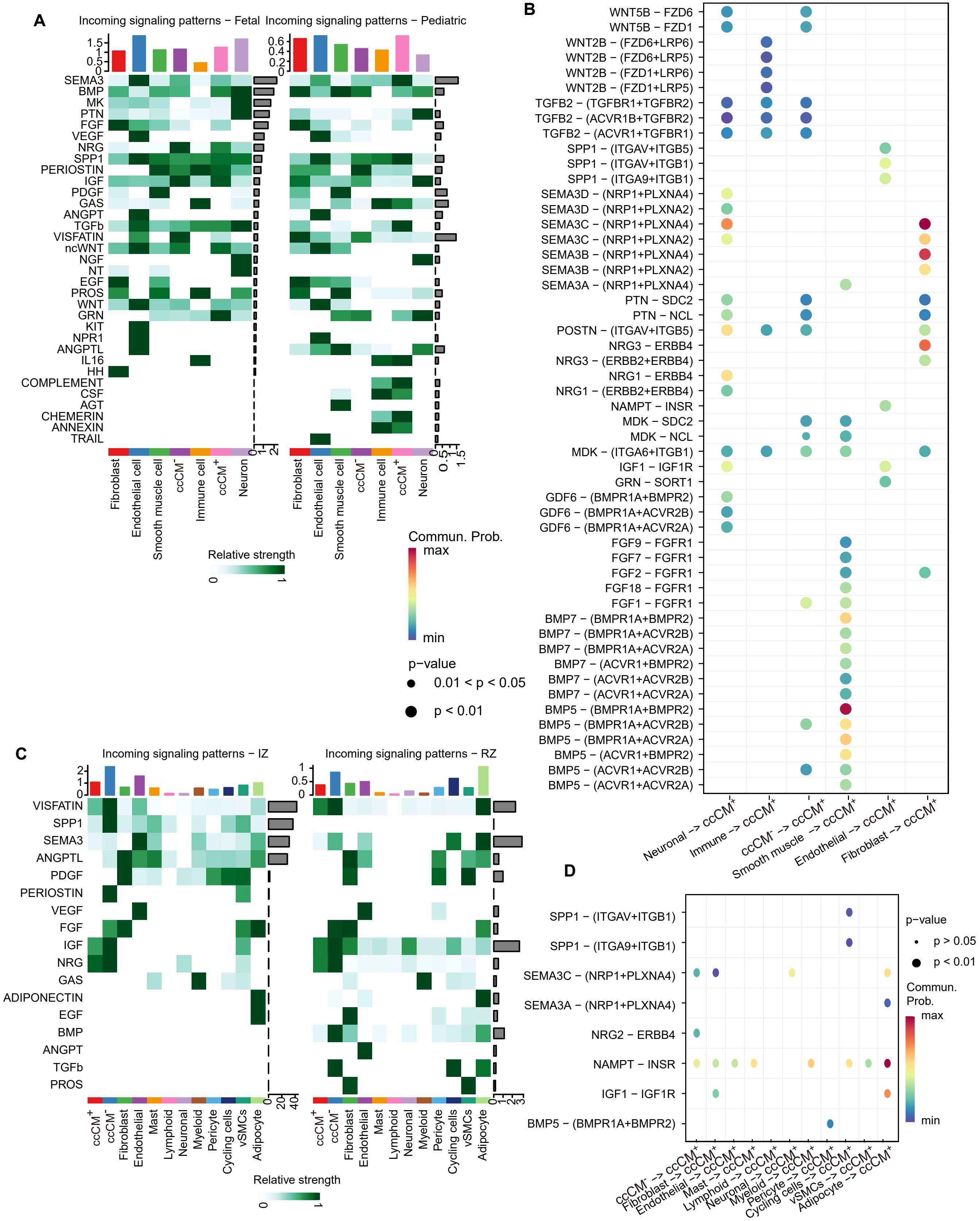

A
Incoming signaling patterns − Fetal
Incoming signaling patterns − Pediatric
SEMA3
BMP
MK
PTN
FGF
VEGF
NRG
SPP1
PERIOSTIN
IGF
PDGF
GAS
ANGPT
TGFb
VISFATIN
ncWNT
NGF
NT
EGF
PROS
WNT
GRN
KIT
NPR1
ANGPTL
IL16
HH
COMPLEMENT
CSF
AGT
CHEMERIN
ANNEXIN
TRAIL
Fibroblast
Endothelial cell
Smooth muscle cell
ccCM-
Immune cell
ccCM+
Neuron
Relative strength
B
WNT5B − FZD6
WNT5B − FZD1
WNT2B − (FZD6+LRP6)
WNT2B − (FZD6+LRP5)
WNT2B − (FZD1+LRP6)
WNT2B − (FZD1+LRP5)
TGFB2 − (TGFBR1+TGFBR2)
TGFB2 − (ACVR1B+TGFBR2)
TGFB2 − (ACVR1+TGFBR1)
SPP1 − (ITGAV+ITGB5)
SPP1 − (ITGAV+ITGB1)
SPP1 − (ITGA9+ITGB1)
SEMA3D − (NRP1+PLXNA4)
SEMA3D − (NRP1+PLXNA2)
SEMA3C − (NRP1+PLXNA4)
SEMA3C − (NRP1+PLXNA2)
SEMA3B − (NRP1+PLXNA4)
SEMA3B − (NRP1+PLXNA2)
SEMA3A − (NRP1+PLXNA4)
PTN − SDC2
PTN − NCL
POSTN − (ITGAV+ITGB5)
NRG3 − ERBB4
NRG3 − (ERBB2+ERBB4)
NRG1 − ERBB4
NRG1 − (ERBB2+ERBB4)
NAMPT − INSR
MDK − SDC2
MDK − NCL
MDK − (ITGA6+ITGB1)
IGF1 − IGF1R
GRN − SORT1
GDF6 − (BMPR1A+BMPR2)
GDF6 − (BMPR1A+ACVR2B)
GDF6 − (BMPR1A+ACVR2A)
FGF9 − FGFR1
FGF7 − FGFR1
FGF2 − FGFR1
FGF18 − FGFR1
FGF1 − FGFR1
BMP7 − (BMPR1A+BMPR2)
BMP7 − (BMPR1A+ACVR2B)
BMP7 − (BMPR1A+ACVR2A)
BMP7 − (ACVR1+BMPR2)
BMP7 − (ACVR1+ACVR2B)
BMP7 − (ACVR1+ACVR2A)
BMP5 − (BMPR1A+BMPR2)
BMP5 − (BMPR1A+ACVR2B)
BMP5 − (BMPR1A+ACVR2A)
BMP5 − (ACVR1+BMPR2)
BMP5 − (ACVR1+ACVR2B)
BMP5 − (ACVR1+ACVR2A)
Neuronal −> ccCM+
Immune −> ccCM+
ccCM- −> ccCM+
Smooth muscle −> ccCM+
Endothelial −> ccCM+
Fibroblast −> ccCM+
Commun. Prob.
max
min
p−value
0.01 < p < 0.05
p < 0.01
C
Incoming signaling patterns − IZ
Incoming signaling patterns − RZ
VISFATIN
SPP1
SEMA3
ANGPTL
PDGF
PERIOSTIN
VEGF
FGF
IGF
NRG
GAS
ADIPONECTIN
EGF
BMP
ANGPT
TGFb
PROS
ccCM+
ccCM-
Fibroblast
Endothelial
Mast
Lymphoid
Neuronal
Myeloid
Pericyte
Cycling cells
vSMCs
Adipocyte
Relative strength
D
SPP1 − (ITGAV+ITGB1)
SPP1 − (ITGA9+ITGB1)
SEMA3C − (NRP1+PLXNA4)
SEMA3A − (NRP1+PLXNA4)
NRG2 − ERBB4
NAMPT − INSR
IGF1 − IGF1R
BMP5 − (BMPR1A+BMPR2)
ccCM- −> ccCM+
Fibroblast −> ccCM+
Endothelial −> ccCM+
Mast −> ccCM+
Lymphoid −> ccCM+
Neuronal −> ccCM+
Myeloid −> ccCM+
Pericyte −> ccCM+
Cycling cells −> ccCM+
vSMCs −> ccCM+
Adipocyte −> ccCM+
p−value
p > 0.05
p < 0.01
Commun. Prob.
max
min

Supplementary Fig. S7 CellChat analysis of intercellular communication networks across developmental and disease contexts. (A) Pathway-level incoming signaling strength to each cell type in fetal (left) and pediatric (right) hearts. (B) Ligand–receptor interactions from indicated sender cell types to $ccCM^+$ in the fetal cohort. Point color encodes communication probability; point size reflects significance. (C) Pathway-level incoming signaling strength in adult IZ (left) and RZ (right). (D) Ligand–receptor interactions from indicated sender cell types to $ccCM^+$ in adult AMI.

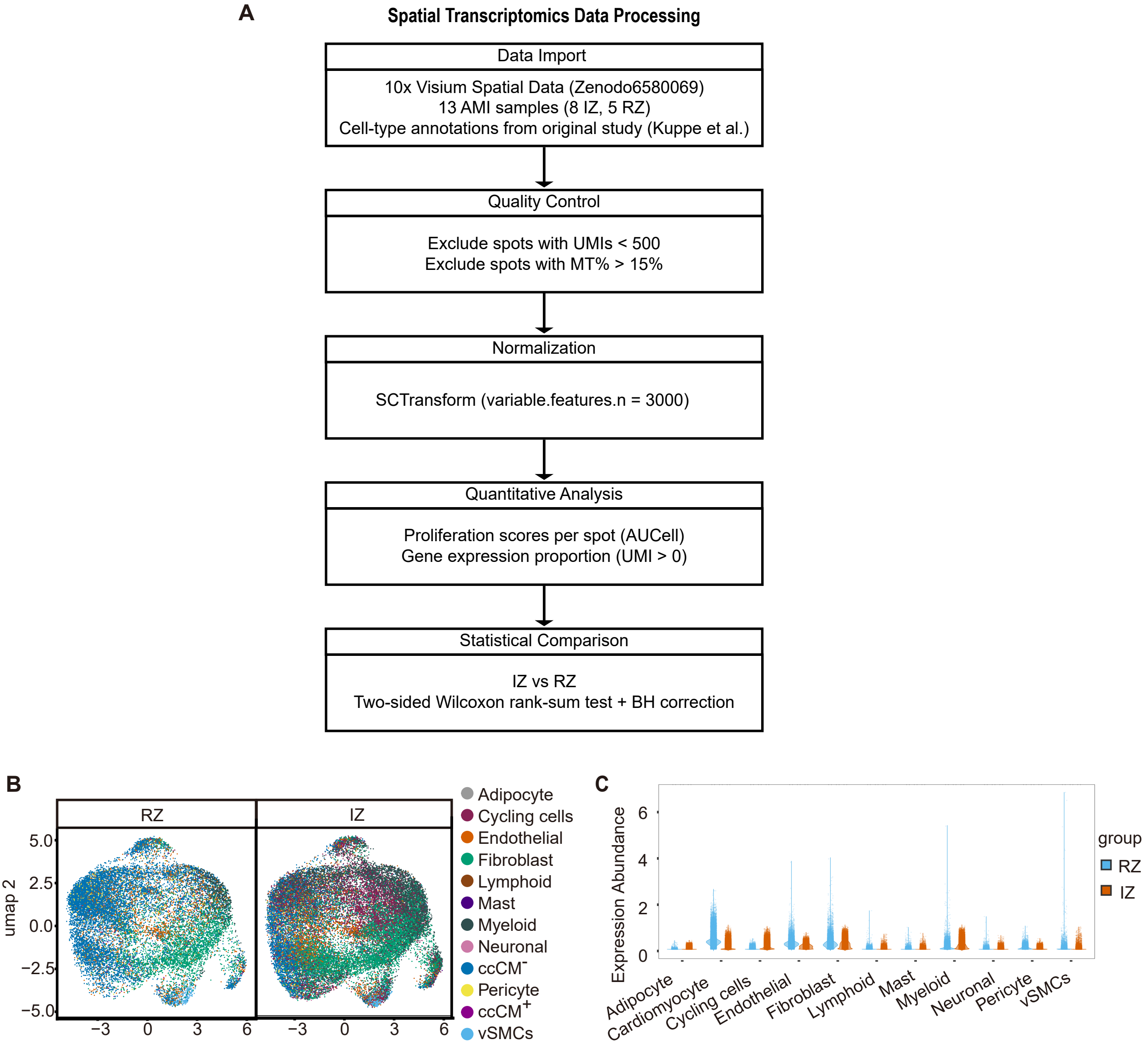
A
Spatial Transcriptomics Data Processing
Data Import
10x Visium Spatial Data (Zenodo6580069)
13 AMI samples (8 IZ, 5 RZ)
Cell-type annotations from original study (Kuppe et al.)
Quality Control
Exclude spots with UMIs < 500
Exclude spots with MT% > 15%
Normalization
SCTransform (variable.features.n = 3000)
Quantitative Analysis
Proliferation scores per spot (AUCell)
Gene expression proportion (UMI > 0)
Statistical Comparison
IZ vs RZ
Two-sided Wilcoxon rank-sum test + BH correction
B
RZ
IZ
umap 2
5.0
2.5
0.0
−2.5
−5.0
−3
0
3
6
Adipocyte
Cycling cells
Endothelial
Fibroblast
Lymphoid
Mast
Myeloid
Neuronal
ccCM⁻
Pericyte
ccCM⁺
vSMCs
C
Expression Abundance
6
4
2
0
Adipocyte
Cardiomyocyte
Cycling cells
Endothelial
Fibroblast
Lymphoid
Mast
Myeloid
Neuronal
Pericyte
vSMCs
group
RZ
IZ

Supplementary Fig. S8 Spatial transcriptomic validation of cellular composition in myocardial infarction tissue. (A) Workflow for spatial transcriptomics data processing (details indicated in the flowchart). (B) UMAP visualization of spatial transcriptomics data, split by RZ and IZ, colored by cell type. (C) Cell-type composition comparing IZ and RZ.

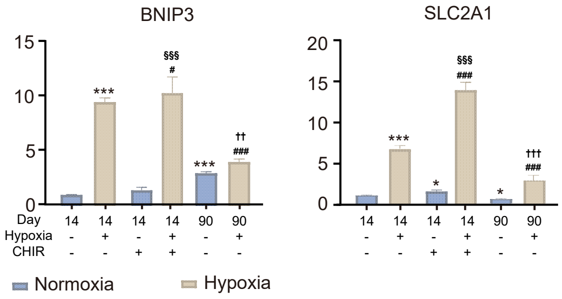

Significance: *vs Day14 Normoxia, #vs Day14 Hypoxia, †vs Day90 Normoxia , §vs Day14 CHIR Normoxia

Supplementary Fig. S9 Validation of the ischemic-hypoxic exposure model in iPSC-derived cardiomyocytes. qPCR analysis of BNIP3 and SLC2A1, canonical HIF-1 target genes, confirming effective hypoxic exposure. Data represent mean ± SEM (n = 8 biological replicates). Two-way ANOVA with Tukey's post-hoc test; significance symbols are defined in the panel.

Supplementary Table Legends

Supplementary Table S1. Developmental dataset information. Summary of human developmental heart samples used in this study, including GEO accession numbers, sample identifiers, developmental stage, age, sex, assay type, cardiac region, and clinical diagnosis. Fetal samples range from 14 to 20 post-conceptional weeks; pediatric samples range from 3 weeks to 14 years. All samples were obtained from left ventricular tissue of individuals without cardiovascular disease. Data were obtained from a previously published cohort (Sim et al., 2021).

Supplementary Table S2. Adult AMI dataset information. Clinical and pathological characteristics of adult AMI and control samples analyzed in this study. Spatial transcriptomics samples (10X and ACH prefixes) and snRNA-seq samples are listed with tissue zone, clinical metadata, and pathology descriptions from the original study. Data were obtained from a previously published cohort (Kuppe et al., 2022).

Supplementary Table S3. qPCR primer sequences. Forward and reverse primers used for qPCR experiments in human iPSC-derived cardiomyocytes. Primer pairs were obtained from PrimerBank (Wang et al., 2012) and were selected to span exon–exon junctions where possible to avoid genomic DNA amplification.
